# Context Matters: Distinct Disease Outcomes as a Result of *Crebbp* Hemizygosity in Different Mouse Bone Marrow Compartments

**DOI:** 10.1371/journal.pone.0158649

**Published:** 2016-07-18

**Authors:** Ting Zhou, Stephanie N. Perez, Ziming Cheng, Marsha C. Kinney, Madeleine E. Lemieux, Linda M. Scott, Vivienne I. Rebel

**Affiliations:** 1 Greehey Children’s Cancer Research Institute, University of Texas Health Science Center at San Antonio (UTHSCSA), San Antonio, TX, United States of America; 2 Department of Cellular and Structural Biology, UTHSCSA, San Antonio, TX, United States of America; 3 Department of Biology, Texas Lutheran University, Seguin, TX, United States of America; 4 Department of Pathology, UTHSCSA, San Antonio, TX, United States of America; 5 Bioinfo, Plantagenet, ON, Canada; 6 University of Queensland Diamantina Institute, and Translational Research Institute, Brisbane, Australia; 7 Cancer Therapy and Research Center, UTHSCSA, San Antonio, TX, United States of America; European Institute of Oncology, ITALY

## Abstract

Perturbations in *CREB binding protein* (*CREBBP*) are associated with hematopoietic malignancies, including myelodysplastic syndrome (MDS). Mice hemizygous for *Crebbp* develop myelodysplasia with proliferative features, reminiscent of human MDS/myeloproliferative neoplasm-unclassifiable (MDS/MPN-U), and a proportion goes on to develop acute myeloid leukemia (AML). We have also shown that the *Crebbp*^*+/-*^ non-hematopoietic bone marrow microenvironment induces excessive myeloproliferation of wild-type cells. We now report that transplantation of unfractionated *Crebbp*^*+/-*^ bone marrow into wild-type recipients resulted in either early-onset AML or late-onset MDS and MDS/MPN-U. In contrast, purified Lin^-^Sca-1^+^c-Kit^++^ cells primarily gave rise to MDS with occasional transformation to AML. Furthermore, *Crebbp*^*+/-*^ common myeloid progenitors and granulocyte/macrophage progenitors could trigger skewed myelopoiesis, myelodysplasia and late-onset AML. Surprisingly, the phenotypically abnormal cells were all of wild-type origin. MDS, MPN and AML can thus all be transferred from *Crebbp*^*+/-*^ BM to wild-type hosts but fractionated bone marrow does not recapitulate the full disease spectrum of whole bone marrow, indicating that not only mutational status but also cellular context contribute to disease outcome. This has important consequences for structuring and interpreting future investigations into the underlying mechanisms of myeloid malignancies as well as for their treatment.

## Introduction

There is substantial evidence that CREB binding protein (CREBBP), a transcriptional co-activator with endogenous acetyltransferase activity [1], plays a pivotal role in suppressing malignant transformation of hematopoietic cells [2]. Translocations disrupting this gene have been found in a proportion of patients with therapy-related and *de novo* myelodysplastic syndrome (MDS) and acute myeloid leukemia (AML) [3–7]. Mutations in its acetyltransferase domain have also been detected in lymphoid malignancies [8–11]. A recent large-scale genomics study of driver mutations in MDS found *CREBBP* abnormalities in a subset of patients [12]. Interestingly, this study also found that defects in genes associated with transcript splicing were early events in the development of MDS. We have previously shown that fetal liver hematopoietic stem cells (HSCs) from *Crebbp*^+/-^ animals differentially express some components of the splicing machinery relative to wild-type (WT) animals and exhibit subtle defects in RNA processing [13]. Furthermore, these animals develop myelodysplastic features within their first year of life [14] and frequently develop hematological malignancies as they age [2, 15].

We have also shown that *Crebbp* hemizygosity compromises the bone marrow (BM) microenvironment's ability to sustain appropriate hematopoiesis [16]. We found that WT BM cells transplanted into *Crebbp*^*+/-*^ animals failed to maintain stem cell numbers and resulted in excessive myeloid differentiation. Intact *CREBBP* function in both stem cells and their niche is thus essential for normal hematopoiesis.

We now report the results of a series of studies in which we transplanted unfractionated or purified subpopulations of *Crebbp*^*+/-*^ BM into WT recipients. The goals of these experiments were to determine whether the hematopoietic abnormalities observed in *Crebbp*^*+/-*^ mice were transferrable and, if so, whether we could pinpoint the cell of origin. We find that *Crebbp*^*+/-*^ marrow harbors transplantable disease and that outcome depends on both the cellular composition of the graft and of the recipient microenvironment. Whole *Crebbp*^*+/-*^ BM gave rise in WT animals to either early-onset AML or to MDS (with or without myeloproliferative neoplasm (MPN)), which occurred later in life. In contrast, the transfer of purified Lin^-^Sca-1^+^c-Kit^++^ cells (LSKs) mostly resulted in MDS, demonstrating that accessory cells play an important role in establishing the myeloproliferation that is part of the phenotype of naïve *Crebbp*^*+/-*^ animals. Moreover, it points to the existence either of distinct initiating cells or of different requirements for the development of MDS/MPN and AML from an identical disease precursor. Finally, transfer of purified *Crebbp*^*+/-*^ myeloid progenitors triggered abnormal development of WT cells, indicating the existence of biologically significant cross-talk between mutant and normal hematopoietic cells in these animals. While revealing unexpected regulatory interactions, our studies provide a model system in which to investigate the interplay between hematopoietic cells bearing *Crebbp* mutations and their environment. More broadly, they present a conceptual framework for dissecting the roles of specific disease genes in MDS, MDS/MPN and AML within a cell type-specific context.

## Materials and Methods

### BM Transplantation Assay

*Crebbp*^*+/-*^ mice [2] were fully back-crossed onto a C57BL/6 background. CD45.1^+^ C57BL/6 mice were originally obtained from the National Cancer Institute (Frederick, MD) and CD45.2^+^ C57BL/6 mice were obtained from Charles River Laboratories (Wilmington, MA). Mice were bred and maintained under pathogen-free conditions at the animal facility at the Greehey Children’s Cancer Research Institute (GCCRI). All animal studies were performed in accordance with the recommendations in the Guide for the Care and Use of Laboratory Animals of the National Institutes of Health and approved by the Institutional Animal Care and Use Committee of the University of Texas Health Science Center at San Antonio (protocol numbers: 06030x, 06131x).

All transplant recipients (WT, CD45.1^+^, 4–6 months old) received two doses of 5.5 Gy, 0.9–1.0 cGy/minute, 5 hours apart (Co^60^ Theratron T-780 unit; Atomic Energy of Canada Limited, Chalk River, Ontario, Canada). Several hours after the last dosing, each recipient was injected with a cell suspension containing the CD45.2^+^ donor cells and a life-sparing dose of 2.0 x 10^5^ unfractionated, CD45.1^+^ WT BM cells (“helper” cells, Fig A in S1 Fig). Transplant recipients were monitored daily for symptoms of ill health (hunching, ruffled fur, reduced mobility and loss of appetite) and were sacrificed immediately when hematological disease was suspected. Monitoring of mice also included a monthly peripheral blood (PB) analysis to detect increased myelopoiesis. Diagnosis was based on the published diagnostic criteria of myeloid hematopoietic neoplasms in mice [17] and human [18–20].

### Fluorescence-Activated Cell Sorting (FACS)

#### PB analysis

PB was collected one month after transplantation and on a monthly basis thereafter to determine donor reconstitution and analyze the mature lineage compartments. Blood from the tail vein was collected, incubated with ammonium chloride solution (NH_4_Cl -150 mM, NaHCO_3_−10 mM, 0.4% EDTA, pH 7.4) for lysis of the red blood cells. Cells were then spun down, resuspended in 2% FBS/PBS and incubated with 0.5 μg purified 2.4G2 anti-Fc receptor (BD Biosciences) for 30 minutes on ice, to block aspecific binding. Samples were divided into two separate tubes and incubated for 30 minutes on ice, one with antibodies to label myeloid cells (0.1 μg/ml PE-conjugated anti-Mac1 and 0.75 μg/ml FITC-conjugated anti-Gr1 antibodies) and the other with antibodies directed against lymphoid cells (0.5 μg/ml FITC-conjugated anti-B220 antibody for B cells and 0.2 μg/ml PE-conjugated anti-CD4 and 0.2 μg/ml PE-conjugated anti-CD8 antibodies for T cells) (BD Biosciences). To confirm donor reconstitution (defined as >1% CD45.2^+^ PB leukocytes containing both myeloid and lymphoid cells) and to track the origin of myeloid and lymphoid cells, 0.5 μg/ml APC-conjugated CD45.2 antibodies and 0.2 μg/ml APC-Cy7-conjugated anti-CD45.1 (BD Biosciences) were added to both samples. The cells were washed twice with 4 ml of 2% FBS/PBS and resuspended in 250 μl 2% FBS/PBS. Five μl 7-Aminoactinomycin D (7AAD) (BD Biosciences) was added to exclude dead cells.

#### BM and spleen analysis

Leukocytes from BM and spleen at the final analysis were stained similarly to the PB staining described above. For the analysis of LSKs and myeloid progenitors in BM [21, 22], cells were first stained with an antibody cocktail, which was a mixture of rat anti-mouse antibodies against lineage markers including CD4, CD8, B220, CD19, Gr1, Ter119 (Invitrogen, Carlsbad, CA), CD3 and Il-7Rα (eBioscience, San Diego, CA). After a blocking step with rat serum (eBioscience) that binds to extra anti-rat IgG, cells were incubated with biotin-conjugated anti-Sca-1, PE-Cy7-conjugated anti-KIT, APC-conjugated anti-CD16/32 (BD Biosciences, San Jose, CA) and FITC-conjugated CD34 (eBioscience) antibodies. Sca-1 staining was visualized using streptavidin-APC-Cy7 (BD Biosciences). The cells were then sorted for HSC-enriched LSKs, common myeloid progenitors (CMPs; Lin^-^Sca-1^-^c-Kit^++^CD16/32^-^CD34^+^ cells) and granulocyte/macrophage progenitors (GMPs; Lin^-^Sca-1^-^c-Kit^++^CD16/32^+^CD34^+^ cells). 7AAD was added to exclude dead cells. Analyses were performed on a FACSCanto (BD Biosciences, San Jose, CA) and with FlowJo software (Tree Star, Inc. Ashland, OR).

Sorting of LSKs and myeloid progenitors followed a similar staining strategy except that after being labeled with the lineage marker cocktail, a negative selection for lineage marker-negative cells (Lin- cells) was first performed by incubation with Dynal sheep anti-rat beads (Dynabeads^®^, Invitrogen, United Kingdom). After depletion, the cells were re-stained with the lineage marker cocktail, followed by PE-conjugated anti-rat IgG (Invitrogen) to visualize the residual lineage positive cells. A FACSAria (BD Biosciences) was used for sorting of the various populations. For details on sorting gates, please see Fig B in S1 Fig.

### Analysis of Hematopoietic Tissues

#### PB analysis

The number of red blood cells (RBC), white blood cells (WBC), and platelets (PLT), as well as differential counts were determined using a VetScan HM2^™^ hematology system (Abaxis, Union City, CA).

#### Cytological and histological analysis

PB smears and spleen touch preparations were stained with Giemsa. Cytological and histological analysis of the BM was performed on Giemsa-stained touch preparations and whole bone sections stained with hematoxylin and eosin or silver stain (to detect reticulin deposition). For the tissue sections, tibias were fixed at 4°C in 10% formalin (Fisher Diagnostic, Waltham, MA) for 36 hours. After decalcification in CalRite solution (Thermo Scientific, Waltham, MA) at 4°C for 15 hours, the bones were stored in 70% ethanol before embedding in paraffin, sectioning and staining. Images were produced at room temperature, using an Olympus BX51 microscope with two oil objectives (UPlanFL N 60x/1.25 and UPlanApo 40x/1.00), an UPlanF1 10x/0.30 objective and a DP72 camera. Cellsens^®^ digital imaging software v.1.3 (www.olympusamerica.com) was used to capture the images.

### Gene Expression Analysis

#### Quantitative RT-PCR

Total RNA was extracted from FACS-purified myeloid cells, obtained from the BM, using the RNeasy Midi Kit according to the manufacturer’s instructions (QIAGEN). RNA reverse-transcription followed, using the High Capacity cDNA Reverse Transcription Kit (Applied Biosciences). All probes used to detect gene expression levels were TaqMan probes (Applied Biosciences). Real-time quantitative RT-PCR was performed with TaqMan Universal PCR Master Mix on the 7500 Real-Time PCR System (Applied Biosciences). Data were analyzed using the ΔΔC_T_ relative quantification [23] and normalized to Gapdh.

#### Nanostring

RNA was isolated from FACS-purified cells using the RNeasy Midi Kit from Qiagen. Samples were then stored at -80°C until ready for shipment to the Genomic and RNA Profiling Core at Baylor College of Medicine (Houston TX) where sample quality checks were performed using the NanoDrop spectrophotometer (Thermo Fisher Scientific) and the Agilent Bioanalyzer. After samples were considered acceptable, multiplex gene expression analysis was performed using the NanoString nCounter Gene Expression platform [24] (NanoString Technologies, Seattle, WA). Briefly, 50 ng of total RNA were hybridized with the NanoString Technologies nCounter GX Mouse Immunology Kit V1 containing 561 unique pairs of 35–50bp reporter probes and biotin-labeled capture probes, including internal reference controls. Overnight hybridization occurred for 17.2 hours at 65°C. Excess probes were removed by a magnetic bead purification step, performed on the nCounter Prep Station (software v v4.0.10.2) on the standard assay. Once unbound probes were washed away, the tripartite structure was bound to the streptavidin-coated cartridge and immobilized. Photobleaching and fluorophore degradation was prevented with the addition of SlowFade. The cartridge containing immobilized samples was transferred to the nCounter Digital Analyzer (software v3.0.1.1) and scanned at 280 FOV. An epi-fluorescent microscope and CCD camera identified sets of fluorescent spots, which were tabulated for data output. Quality control metrics were recorded using the nSolver Analysis Software v1.1 (NanoString Technologies). Bioinformatic analysis of the data was performed using methodology described by Brumbaugh *et al*. [24] Samples and genes were clustered in an unsupervised hierarchical matter.

### MMP9 Western Blot Analysis

MMP9 protein levels were measured in whole cell lysates (Abcam, Cambridge, MA), using ACTINB as a control (Sigma, St. Louis, MO) [16]. Primary antibodies were detected with an HRP-coupled secondary antibody and visualized by chemiluminescence (Thermo Fisher Scientific, Rockford, IL). ImageJ software Version 1.43 was used to quantify the Western blot.

### Statistical Analysis

Significant differences between two groups were determined by two-tailed T-tests or Wilcoxon-Mann-Whitney tests, as appropriate. Prism5 (GraphPad Software, Inc., La Jolla, CA) was used to measure differences in survival. As data were not censored, the p-value from the Gehan-Breslow-Wilcoxon Test, which weights early deaths more heavily, is reported in the text. p<0.05 was considered statistically significant.

## Results

### WT Mice Develop Early-Onset AML or Late-Onset MDS after Transplantation with Unfractionated *Crebbp*^+/-^ BM Cells

**Fig 1:**
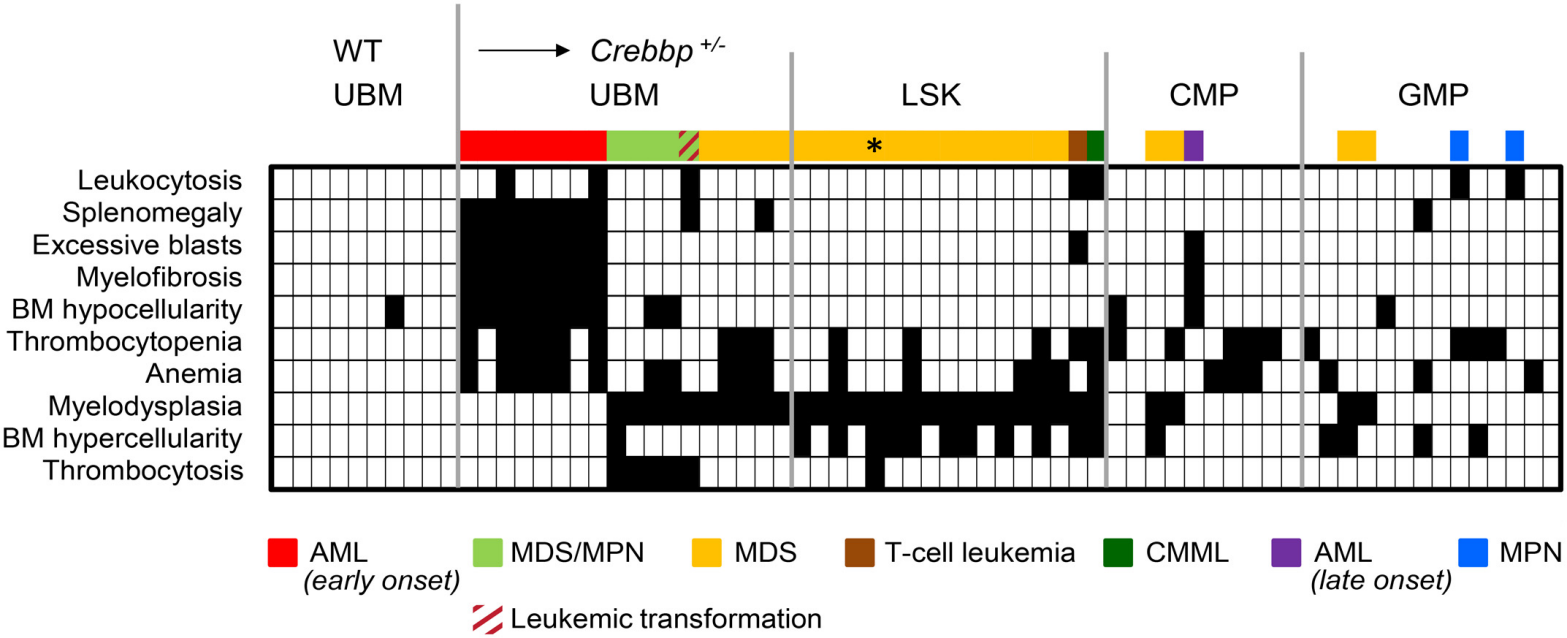
Summary of disease outcomes in WT recipients of tested BM populations. Block plot showing the features and diagnoses (rows) for each of the transplanted animals (columns). White blocks indicate the absence and black blocks the presence of each of the features listed. For leukocytosis, thrombocytopenia, anemia, BM hypercellularity and thrombocytosis to be called present, the measured value had to be 2 SD above or below the mean of the WT group. For splenomegaly, a 4-fold cut-off was chosen because in humans a 3–4 fold-size increase represents a “palpable splenomegaly” [25], while BM hypocellularity was defined as <25% of the mean WT value [26]. Disease outcome for each mouse is color-coded above the block plot. Corresponding disease states are indicated below the plot. * Although this mouse presented with thrombocytosis in addition to >10% myelodysplasia, it was not diagnosed as MDS/MPN; it was sacrificed approximately 6 months earlier than the other mice due to a worsening chronic eye infection. This eye inflammation is likely the cause of the moderate leukocytosis (<2SD) and thrombocytosis.

To test whether the MDS/MPN-U and AML that develop in *Crebbp*^+/-^ mice are transferable, we transplanted *Crebbp*^+/-^ BM cells into WT irradiated recipients. We expect 100% of the *Crebbp*^+/-^ mice older than 1 year to have developed MDS [14], and approximately 40% to harbor leukemogenic cells [2]. To maximize the likelihood that donor BM contained MDS and/or leukemia-initiating cells, we harvested and pooled BM from 8–10 *Crebbp*^+/-^ mice aged between 18–24 months. Four different pools of BM cells were used in 4 independent experiments (S1 Table). Irradiated animals were injected with 4–5 x 10^6^ unfractionated BM cells; as controls, an equivalent dose of unfractionated BM from pools of 3–4 age-matched, WT mice were transplanted into a separate group. Disease development in the various transplantation groups are summarized in Fig 1.

**Fig 2:**
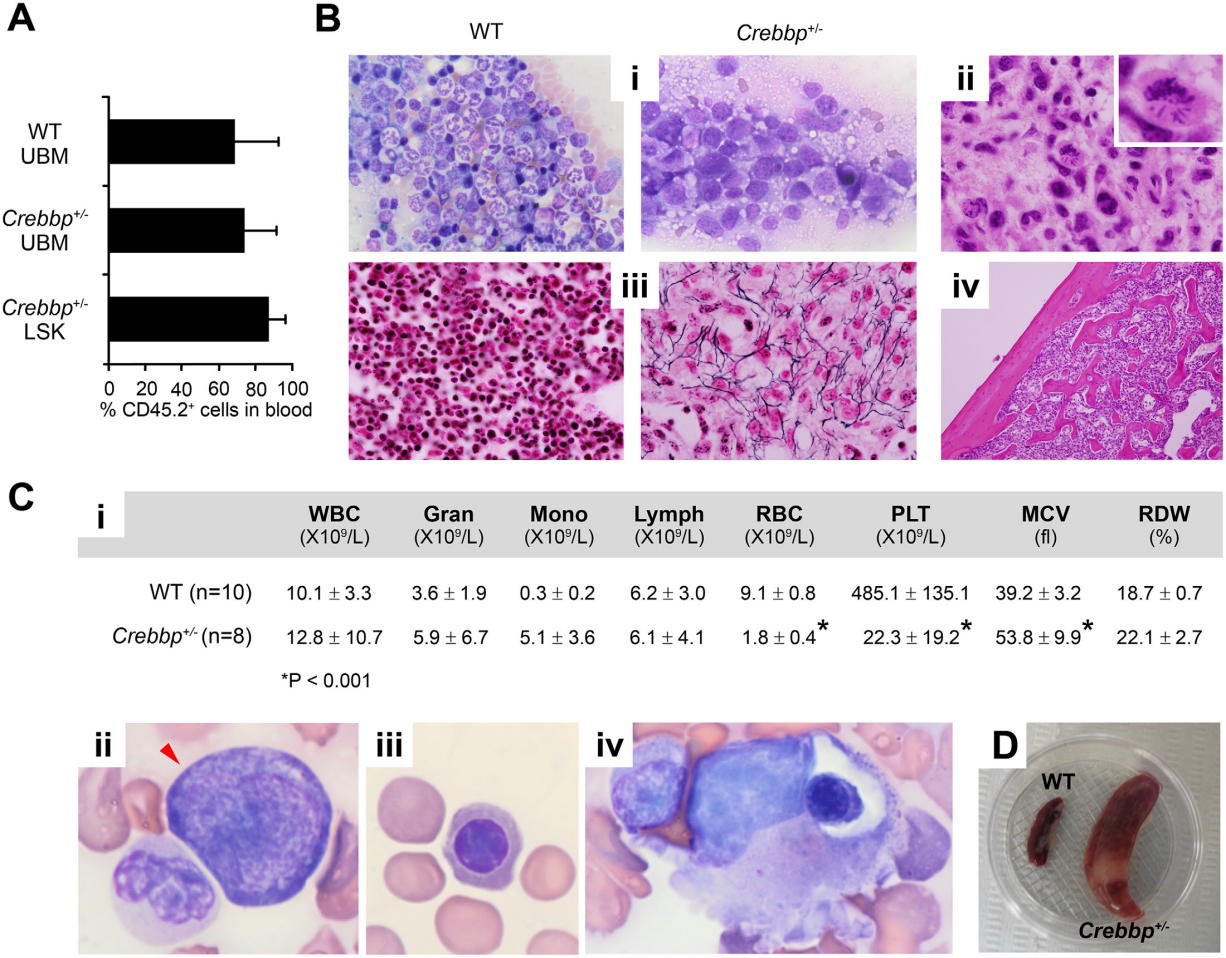
AML and myelofibrosis in WT mice transplanted with unfractionated *Crebbp*^*+/-*^ BM. (**A**) Average percentage + SD of donor-derived (CD45.2^+^) PB leukocytes at the time of sacrifice in recipients transplanted with unfractionated (U) WT or *Crebbp*^+/-^ BM or with *Crebbp*^+/-^ LSK, as indicated. Data were pooled from 3–4 independent experiments. See S1 Table for details. (**B**) Representative histological images of BM touch preparations (**i**) and bone sections from the tibia (**ii-iv**) (**i**) WT control (left) showing abundant erythroid precursors and mature segmented granulocytes. *Crebbp*^*+/-*^ bone section (right) showing dramatically decreased erythroid precursors and the presence of >20% blasts. (**ii**, inset) *Crebbp*^*+/-*^ bone section, showing an atypical mitotic figure. (**iii**) Reticulin stain of bone sections; WT control (left) and *Crebbp*^*+/-*^ BM (right) demonstrating in the latter a marked increase in reticulin (black fibers). (**iv**) *Crebbp*^*+/-*^ bone section showing an increase in and thickening of trabeculae. (C) (**i**) Blood cell counts showing the average ± SD of the indicated parameters in recipients of *Crebbp*^*+/-*^ BM that developed AML and WT controls. * p<0.001. (**ii-iv**) Circulating blasts (**ii**), nucleated RBCs (**iii**) and RBC phagocytosis (**iv**). (**D**) Spleen from a recipient of *Crebbp*^*+/-*^ BM and a WT control. Original magnification: ×60 (Bi, Bii), ×40 (Biii), ×10 (Biv).

Reconstitution levels achieved by unfractionated *Crebbp*^*+/-*^ BM cells were similar to that of WT cells (Fig 2A and Fig C in S1 Fig). As expected, recipients of WT cells did not develop any hematological disorders (n = 10), while all 18 recipients receiving unfractionated *Crebbp*^*+/-*^ BM became ill. In total, 8/18 developed AML early, within 2–7 months, while the other 10 developed either MDS/MPN-U or MDS with a longer latency of 12–18 months (Fig 1). The AMLs were characterized by a BM histology showing a uniform population of blast cells (>20%) replacing normal trilineage hematopoiesis (Fig 2Bi, compare WT and *Crebbp*^*+/-*^) and an increase in mitotic figures (Fig 2Bii and inset). The leukemias were accompanied by extensive myelofibrosis not present in WT (Fig 2Biii) with increased trabecular bone formation (Fig 2Biv). BM cellularity was consequently extremely low (13.6 ±11.7 x 10^6^ cells/2 femurs compared to 43.7 ±8.6 x 10^6^ in WT controls, p<0.001). The BM hypocellularity may explain the absence of the leukocytosis that is typically a hallmark of AML. The PB mirrored the loss of erythroid precursors noted in the marrow and furthermore showed features that often accompany myelofibrosis in humans, such as extreme thrombocytopenia (Fig 2Ci), circulating blast cells (Fig 2Cii, arrow head) and damaged and phagocytosed red cells (Fig 2Ciii and 2Civ) [27, 28]. The spleens also were significantly enlarged (Fig 2D). Flow cytometric analysis of the BM, PB and spleen revealed the presence of a large, uniform population of cells that stained positive for Mac1 and Gr1, indicating that the leukemia in these mice was of myeloid origin (S2 Fig).

One animal progressed to leukemia 16 months after transplantation (S3 Fig). CBC showed leukocytosis and thrombocytosis while histologic examination of the PB and the BM revealed an accumulation of cells with a lymphocytic morphology (Fig A in S3 Fig). Flow cytometric analysis demonstrated the expression of lymphoid specific markers, confirming the lymphoid origin of the tumor, as well as low levels of the myeloid cell marker Gr1. The co-expression of lymphoid and myeloid expression is commonly observed in human lymphocytic leukemia [29] (Fig B in S3 Fig). In contrast to the animals that developed early-onset leukemia, this mouse also exhibited features of myelodysplasia (Figs Aii and C in S3 Fig), suggesting that the leukemia was likely proceeded by MDS/MPN.

**Fig 3:**
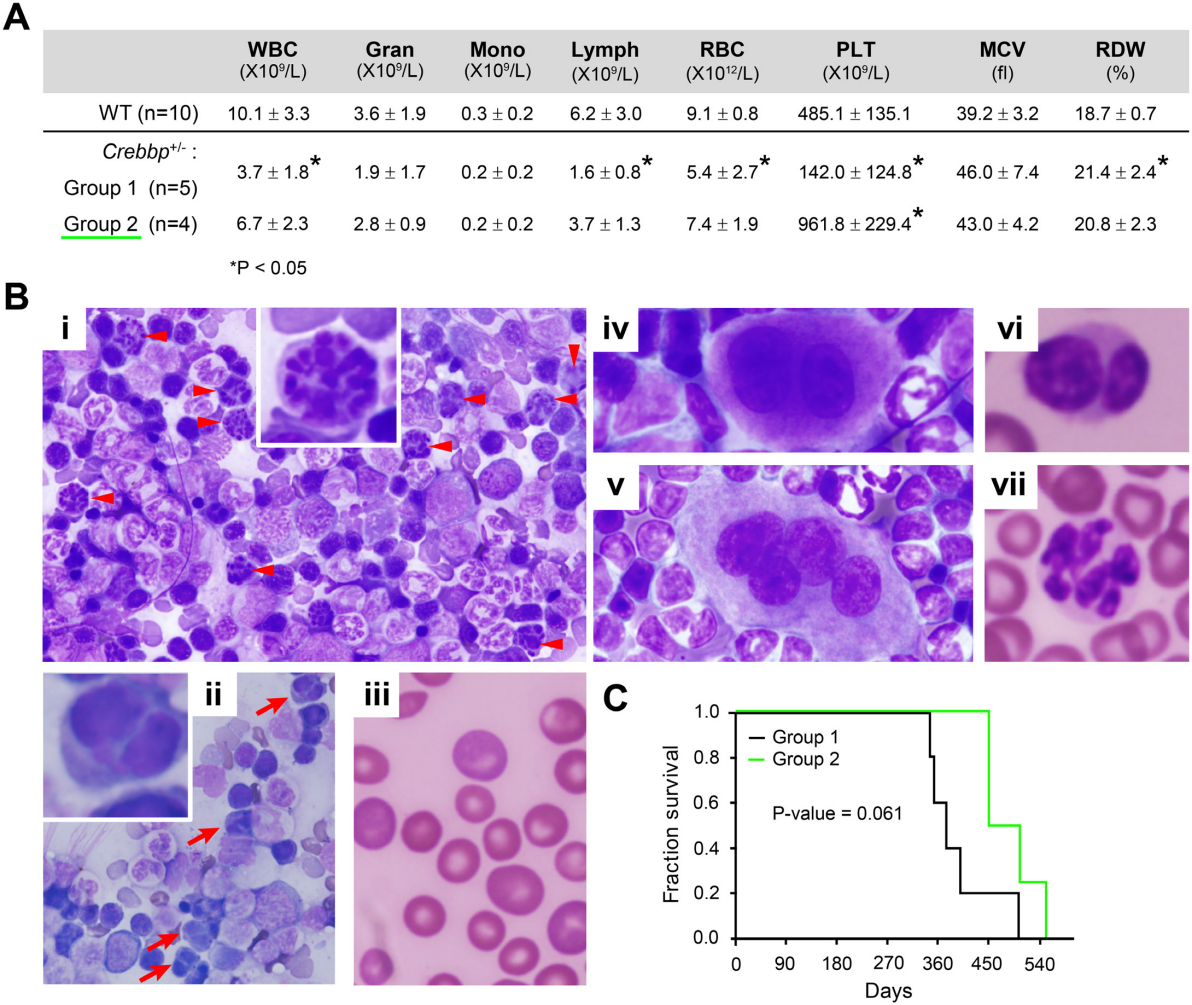
Hematologic characteristics of later-onset myelodysplastic disease in recipients of unfractionated *Crebbp*^+/-^ BM. (**A**) Presented are averages ± SD of WBCs, RBCs, PLTs and spleen weights. Recipients were divided into two groups based on the absence (Group 1) or presence (Group 2) of thrombocytosis, the determining factor for a diagnosis of MDS vs. MDS/MPN-U. * p<0.05 compared to WT control. (**B**) BM touch preparations (**i-ii** and **iv-v**) and PB smears (**iii** and **vi-vii**) showing characteristic features of tri-lineage myelodysplasia. (**i-iii**) Dysplasia of erythroid lineage manifested by karyorrhexis (**i**, inset and arrow heads) i.e., segmentation of the red cell nucleus and binucleated erythroid precursors (**ii**, inset and red arrows) as well as anisocytosis (**iii**). (**iv-v**) Dysplasia of megakaryocytic lineage indicated by binucleated dwarf megakaryocytes (**iv**) and multinucleated megakaryocytes (**v**). (**vi-vii**) Dysplasia of myeloid cells with pseudo Pelger-Huet anomalies (**vi**) and hypersegmented granulocytes (i.e. with more than six segments of irregular size) (**vii**). Original magnification: ×40 (**i-ii**), ×60 (**iii-vii**). (**C**) Survival curve of recipients of unfractionated *Crebbp*^+/-^ BM transplants.

The hematopoietic disease observed in the 9 remaining recipients displayed strikingly different features (Fig 1). All showed myelodysplasia, but could be further subdivided into two groups based on the absence (Group 1) or presence (Group 2) of an accompanying thrombocytosis (Fig 3A). Both groups displayed mild splenomegaly (0.26 ±0.11 g and 0.23 ±0.10 g, respectively, compared to 0.10 ±0.00 g for WT controls) and a normocellular BM (10^6^ cells/two femurs: Group 1 = 44.2 ±4.5, Group 2 = 43.9 ±20.6, and WT = 43.7 ±8.6). Moreover, a tri-lineage dysplasia in >10% of the cells in each affected lineage was observed (Fig 3B). The presence of significant thrombocytosis and the absence of clear cytopenia in other lineages warranted the diagnosis of MDS/MPN-U [20] for Group 2, as opposed to MDS for Group 1. Interestingly, Group 1 mice had severe pancytopenia and died appreciably earlier than those in Group 2 (Fig 3C). Group 1 animals showed a relative increase in mature Mac1^+^Gr1^+^ granulocytes (S4 Fig); however, this was insufficient to warrant a classification of granulocytosis.

### Transplantation of Purified *Crebbp*^+/-^ LSK Cells Causes MDS but Not Early-Onset Leukemia

To determine whether the long-term repopulating BM fraction harbored any or all of the disease-initiating cells, LSK cells were isolated from 3 of the same pooled *Crebbp*^+/-^ cell suspensions as above and transplanted into separate groups of WT recipients (Fig A in S1 Fig). The number of purified cells transplanted per recipient was calculated so that each recipient received a dose equivalent to that contained within the unfractionated BM transplant. The calculation was based on the frequency of each sorted population in the BM, which was determined by FACS (S2 Table). In total, 17 WT recipients each received 1.0–2.5 x10^4^ *Crebbp*^*+/-*^ LSK cells resulting in a reconstitution level comparable to that of 5 x 10^6^ unfractionated WT or *Crebbp*^+/-^ BM cells (Fig 1A and Fig C in S1 Fig). Strikingly, unlike recipients of unfractionated BM, none of the LSK recipients developed an early-onset leukemia (Fig 1) (binomial p-value = 4.6 x 10^−5^ given the 8/18 proportion observed with unfractionated BM).

Two recipients in this cohort died from late-onset leukemia, 14 months after transplantation. One showed a disease resembling human mature T-cell leukemia, characterized by extreme leukocytosis (109.6 x10^9^/L) due to increased numbers of Gr1^lo^CD8^+^ T cells, anemia, thrombocytopenia, mild splenomegaly and enlarged lymph nodes [29] (Figs A and B in S5 Fig). The second mouse displayed a disease reminiscent of human chronic myelomonocytic leukemia (CMML), with tri-lineage myelodysplasia, granulocytosis (42.0 x 10^9^/L), monocytosis (6.6 x10^9^/L), anemia, thrombocytopenia and mild splenomegaly. Histologically, both the PB and BM showed a dominance of well-differentiated mature myeloid cells (Figs C-E in S5 Fig).

**Fig 4:**
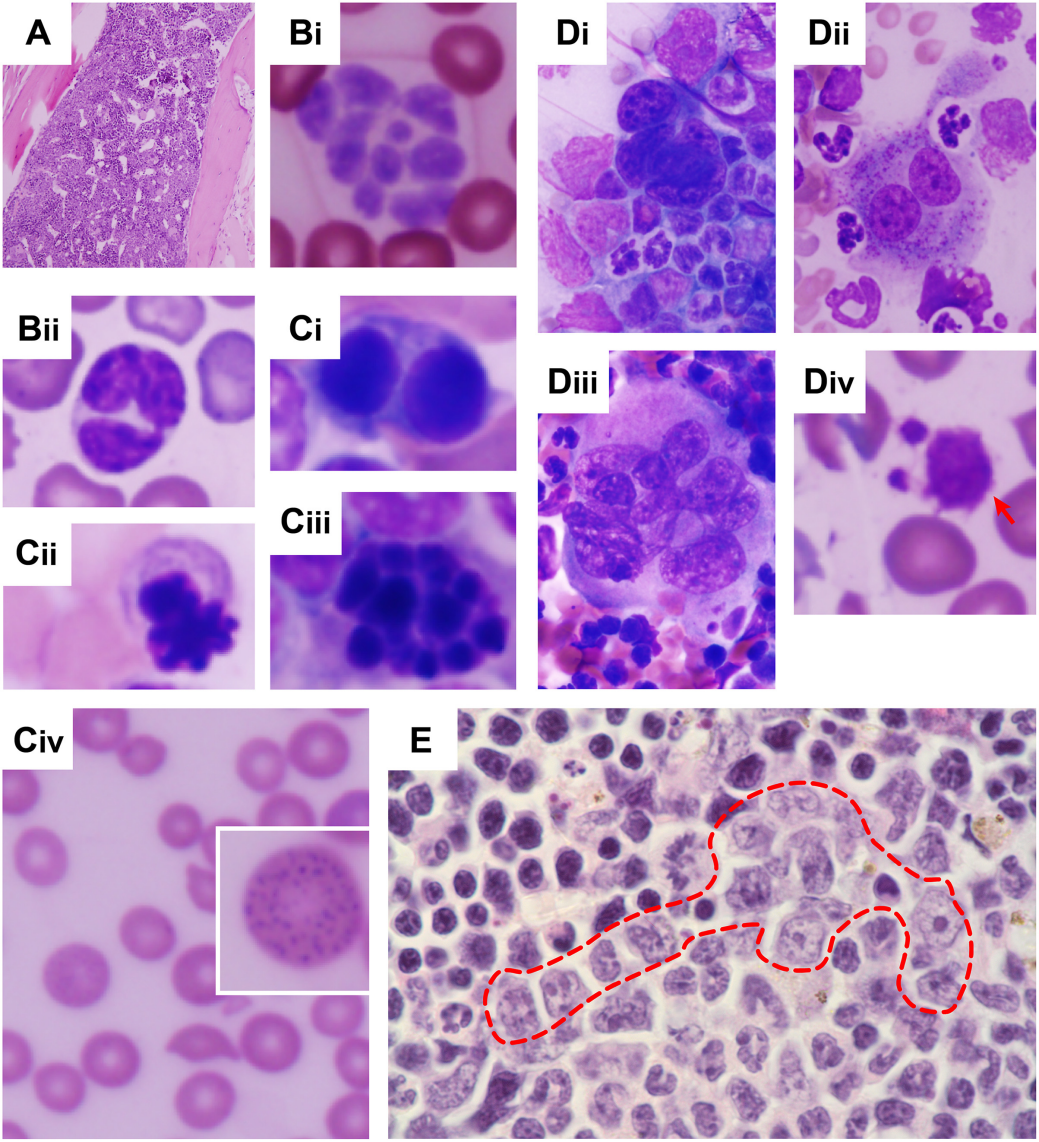
Hypercellular BM and tri-lineage myelodysplasia in WT mice transplanted with *Crebbp*^+/-^ LSKs. (**A**) BM section showing a hypercellular BM. (**Bi-ii, Civ, Div**) PB smears and (**Ci-iii, Di-iii**) BM touch preparations showing characteristics of tri-lineage myelodysplasia. Dysplasia of the myeloid lineage is indicated by hypersegmented granulocytes (**Bi**) and pseudo Pelger-Huet anomalies (**Bii**). Dysplasia of the erythroid lineage was often manifested by binucleation (**Ci**), an abnormal nuclear contour (**Cii**) and karyorrhexis (**Ciii**). Anisocytosis (**Civ**) and basophilic stippling (**Civ**,inset) are other features of pathological erythropoiesis found in these mice. Dysplasia of the megakaryocytic lineage is characterized by naked megakaryocytic nuclei (**Di**), binucleated megakaryocytes D**ii**), hyperlobulated megakaryocytes (**Diii**) and giant PLTs (**Div**,arrow). (**E**) Atypical localization of immature precursors (i.e., clusters of myeloid precursors present in the intertrabecular area (red dashed line), rather than adjacent to trabeculae or surrounding endothelial cells. Magnification: ×10 (**A**), ×40 (**Civ**), ×60 (**B**, **Ci-iii**, **D-E**).

The 15 remaining mice developed MDS between 11 and 17 months after transplantation. The disease in these animals was characterized by ineffective hematopoiesis, as evidenced by anemia (hemoglobin: 11.3 ±2.3g/dL) and marrow hypercellularity (66.5 ±17.5 x10^6^ cells/two femurs; Fig 4A). The BM showed unmistakable signs of dysplasia in >10% of myeloid (Fig 4Bi and 4Bii), erythroid (Fig 4Ci and 4Civ) and megakaryocytic cells (Fig 4Di and 4Div). In some instances, atypical localization of immature precursors, a feature in humans consistent with MDS or early leukemogenesis [14], was also apparent (Fig 4E). Intriguingly, *Crebbp*^+/-^ LSK recipients did not have an obvious myeloproliferative phenotype, such as the granulocytosis or thrombocytosis observed in naïve *Crebbp*^+/-^ mice or in ~25% of unfractionated *Crebbp*^+/-^ BM transplants.

### Transplanted *Crebbp*^+/-^ CMPs or GMPs Induce Abnormalities in WT Hematopoietic Cells

**Fig 5:**
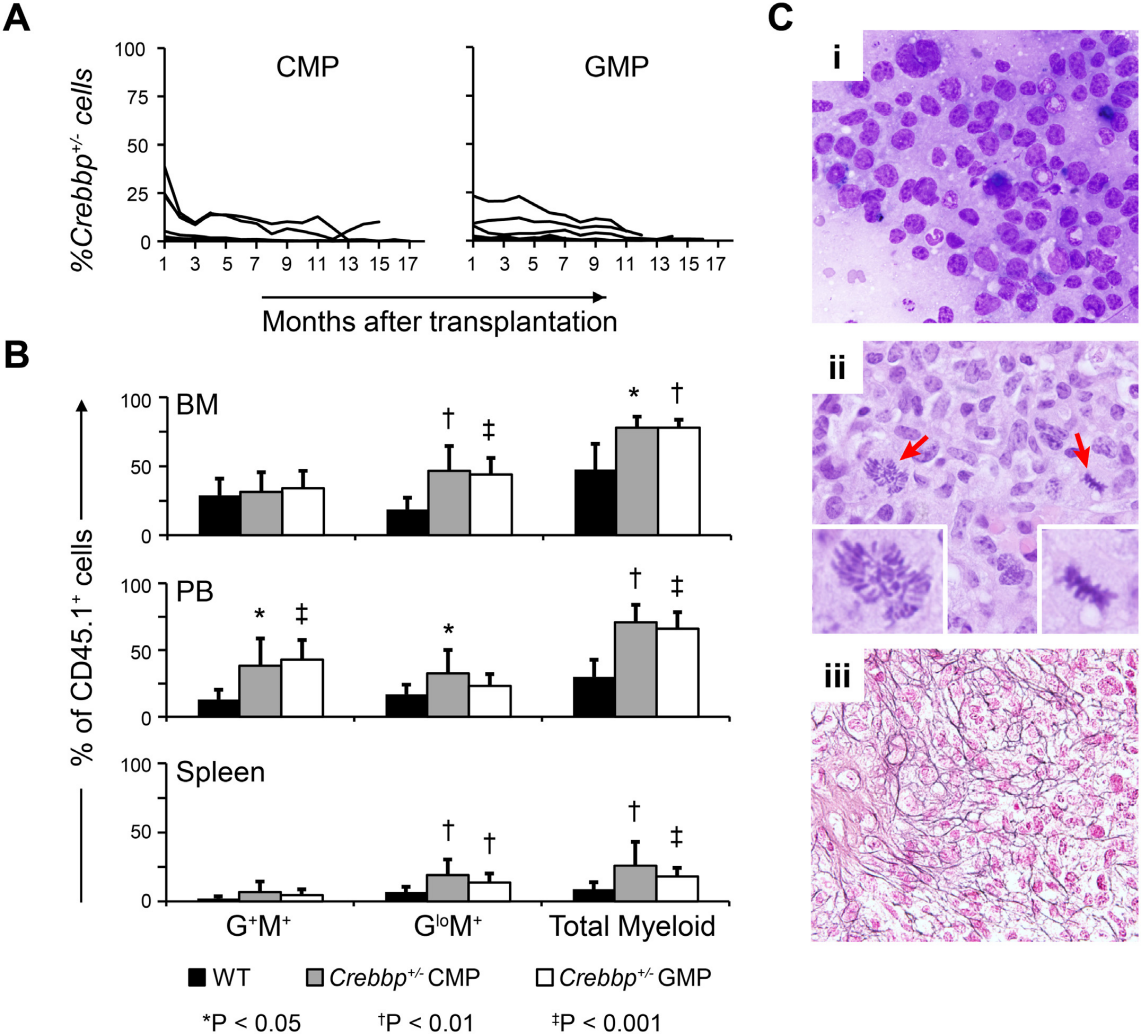
Increased myelopoiesis of WT origin in WT mice transplanted with *Crebbp*^+/-^ CMPs or GMPs. (**A**) Proportion of *Crebbp*^*+/-*^ cell-derived reconstitution (CD45.2^+^) in the PB of individual WT recipients (CD45.1^+^) as a function of time. Left panel shows 10 recipients of *Crebbp*^*+/-*^ CMPs, right panel shows 14 recipients of *Crebbp*^*+/-*^ GMPs. (**B**) Bar graphs showing the average percentage + SD of (CD45.1^+^) WT myeloid populations in the BM (upper panel), PB (middle) and spleen (bottom). Black bars represent recipients transplanted with CD45.2^+^ WT BM, gray bars recipients of CD45.2^+^ *Crebbp*^*+/-*^ CMPs and white bars recipients of CD45.2^+^ *Crebbp*^*+/-*^ GMPs. Measurements were taken at the time of sacrifice, i.e., between 10–19 months (S3 Table). Symbols indicate significant differences with WT; * p<0.05; ^†^ p<0.01; ^‡^ p<0.001. (**C**) Histological evidence of AML in mouse #5 (S3 Table). (**i**) BM touch preparation showing the prevalence of blasts (>20%) and the relative lack of maturing trilineage hematopoiesis. (**ii**) H&E-stained BM section. Arrows and inserts indicate an atypical mitotic figure. (**iii**) Reticulin staining of BM section. Magnification: ×60 (**A-B**) and ×40 (**C**).

It is well known that certain myeloid populations have tumor-promoting capabilities [30–37] and that introduction of certain oncogenes into murine myeloid progenitor cells can give rise to leukemia [38–41]. We therefore tested whether CMPs or GMPs could function as the missing early-onset AML-initiating cells. Aliquots of FACS-purified *Crebbp*^+/-^ CMPs or GMPs were isolated from 2 of the same pools of *Crebbp*^+/-^ BM as before and transplanted into 10 or 14 WT recipients, respectively, along with 2 x 10^5^ CD45.1^+^ helper cells. From the outset, *Crebbp*^*+/-*^ donor cell-derived engraftment was low for most recipients and declined over time. At the time of sacrifice, few CD45.2^+^ cells were detectable in the PB, BM (Fig 5A, S3 and S4 Tables) or spleen (data not shown). Importantly, the BM of the *Crebbp*^+/-^ CMPs and GMPs also did not harbor any *Crebbp*^+/-^ LSKs (S6 Fig and S3 Table).

None of the *Crebbp*^+/-^ CMP or GMP recipients developed early-onset leukemia (combined p-value = 7.5 x 10^−7^ of being the same frequency as whole *Crebbp*^+/-^ BM). Surprisingly, despite the low levels of hematopoietic reconstitution by transplanted CMPs and GMPs (Fig 5A), FACS analysis revealed an increased proportion of myeloid cells of WT (CD45.1^+^) origin relative to animals receiving only WT cells (Fig 5B). Moreover, 19/24 (79%) mice that received *Crebbp*^+/-^ progenitors showed evidence of hematopoietic abnormalities at the time of sacrifice (Fig 1). In particular, dysplasia in one or more lineages was found in 13 animals (S3 Table). In 2/10 recipients of *Crebbp*^*+/-*^ CMPs and in 2/14 recipients of *Crebbp*^*+/-*^ GMPs, the level of dysplasia was present in >10% of a particular lineage, warranting a diagnosis of MDS [18]. Two other GMP recipients were diagnosed with MPN (recipients #16 and #24 in S3 Table) based on the observation that they displayed leukocytosis, but did not have myelodysplasia [17]. Finally, one CMP recipient was diagnosed with AML 11.6 months post-transplant (recipient #5 in S3 Table). Its BM contained >20% myeloblasts, with virtually no erythroblasts or megakaryocytes (Fig 5Ci). Consistent with the diagnosis of AML, mitotic cells and polyploidy were readily apparent within the marrow (Fig 5Cii). Extreme hypocellularity (2.9 x 10^6^ cells/2 femurs) and positive BM reticulin staining (Fig 5Ciii), in addition to an enlarged spleen (0.3 g), indicated extensive myelofibrosis.

Transplantation with small numbers of stem cells results in considerable proliferative stress in the marrow of recipients [42]. To test whether the abnormalities we observed in the CMP and GMP recipients resulted from an endogenous response to this stress, the same number of unfractionated CD45.2^+^ WT BM cells (2 x 10^5^) were transplanted into irradiated CD45.1^+^ WT hosts, but now without *Crebbp*^*+/-*^ CMPs or GMPs. Recipients were monitored for 15 months for disease development. At sacrifice, 75% of hematopoietic cells were CD45.2^+^ and the relative level of myeloid reconstitution was similar to that found in the recipients of 5 x 10^6^ unfractionated WT BM cells described earlier. Most important, no evidence of myelodysplasia, MDS or other overt hematological disorder was detected (S7 Fig).

### Mature *Crebbp*^+/-^ Myeloid Cells Overexpress MMP9 and Have Altered Cytokine and Gene Expression Profiles

**Fig 6:**
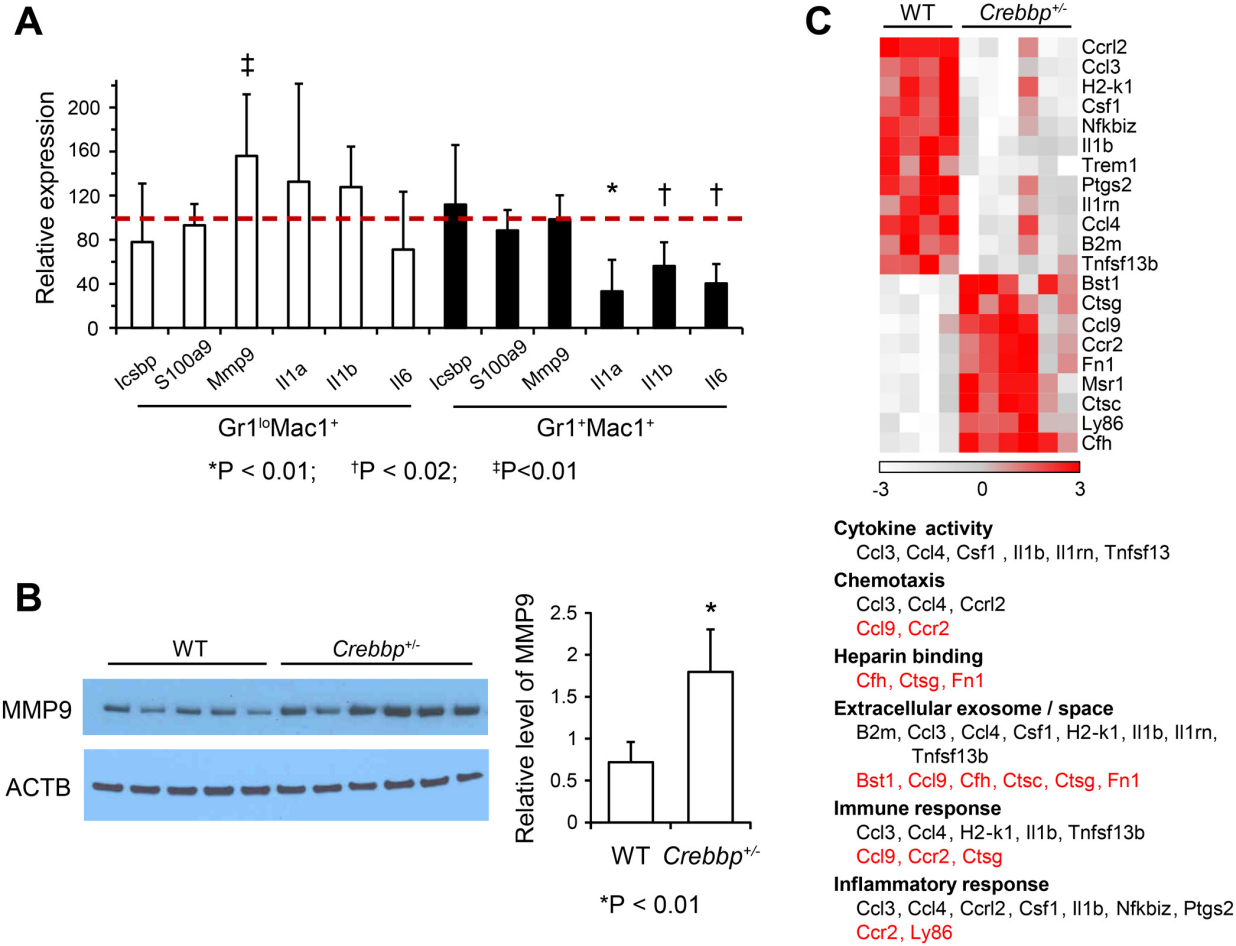
Mature *Crebbp*^*+/-*^ myeloid cells produce excess MMP9 and have altered cytokine and gene expression profiles. (**A**) Quantitative RT-PCR analysis for genes found differentially expressed in tumor-promoting myeloid cells or in MDS patients. Presented is the ratio of gene expression in *Crebbp*^*+/-*^ myeloid populations (n = 7) compared to WT controls (n = 6), purified from the BM of 1 year-old animals. Symbols indicate statistical significance; * p<0.05; ^†^ p<0.02; ^‡^ p<0.01. (**B**) MMP9 Western Blot analysis (left panel) performed on Gr1^lo^Mac1^+^ cells and corresponding quantification (right panel, average + SD). (**C**) Heat map of transcripts of the NanoString Immunology Panel differentially expressed (adjusted P-value < 0.05) in *Crebbp*^*+/-*^ vs WT Gr1^+^Mac1^+^ cells. The table below the heat map represents the gene ontology terms overrepresented versus the platform background and the associated genes. Genes in grey are downregulated; those in red, upregulated.

Gr1^+^Mac1^+^ cells, the mature progeny of CMPs/GMPs, have been shown to promote the development of malignancies, including MDS [30–37]. Given the hematological abnormalities induced in WT BM by the presence of *Crebbp*^*+/-*^ CMPs/GMPs, we wondered whether such tumorigenic myeloid cells (TMC) might be at play in our system. We therefore isolated Gr1^+^Mac1^+^ cells and their precursors, Gr1^lo^Mac1^+^ cells, from 12 month-old *Crebbp*^*+/-*^ mice, an age by which the animals invariably have MDS. We first established the transcript levels of S100a9, Icsbp (Irf8) and Mmp9, which are known to be expressed at aberrant levels in TMC [30, 32, 34, 35, 37]. S100a9 and Icsbp levels were normal in both populations but MMP9 mRNA and protein levels were significantly increased in *Crebbp*^+/-^ Gr1^lo^Mac1^+^ precursors as compared to WT (Fig 6A and 6B). We also analyzed both populations for mRNA expression levels of Il1a, Il1b, Il6, and Tnf (TNFa), cytokines known to be altered in TMC [32, 34, 35]. *Crebbp*^+/-^ Gr1^+^Mac1^+^ cells produced significantly lower levels of Il1a, Il1b, and Il6 than their WT counterparts (Fig 6A) while Tnf levels were unchanged (data not shown). TMC have also been reported to trigger a decline in immunosurveilance [32, 34, 35] and excessive inflammation through overproduction of pro-inflammatory cytokines [37]. A focused analysis of 561 immunology-related transcripts using Nanostring probes confirmed reduced levels of the Il1b transcript. Il1a and Il6 levels were unchanged using this platform though the discrepancies could be attributed to probe design details. This analysis also revealed additional significant differences (Fig 6C and S5 Table). Together these data suggest that *Crebbp*^+/-^ mature myeloid cells could transmit abnormal signals to neighboring cells, whether by increased release of MMP9 [30], through increased heparin binding [43, 44], disturbed cytokine signaling [32, 34, 35], or altered extracellular exosome/protein signaling [34–37, 45].

## Discussion

We have extensively characterized the hematopoietic system of mice carrying a single functional *Crebbp* allele in previous studies [2, 14, 16]. We now report that the hematopoietic abnormalities of *Crebbp*^*+/-*^ mice can be transferred by the transplantation of unfractionated or purified BM cells into WT hosts, albeit with distinct kinetics (summarized in Fig 1). Roughly half the recipients of unfractionated BM developed AML within 2–7 months while the other half developed MDS or MDS/MPN-U with a longer latency (12–18 months). Interestingly, naïve *Crebbp*^*+/-*^ mice developed MDS/MPN-U around 9–12 months of age, which progressed to AML in ~40% of the cases [2, 14]. The delay in the appearance of MDS in transplanted WT mice relative to naïve *Crebbp*^*+/-*^ animals suggests that a CREBBP deficient microenvironment, which is present in naïve mice but absent in the transplantation model, contributes to the development of hematopoietic diseases in these animals. It is conceivable that the dysfunctional environment needs to be recreated in the transplanted mice before MDS can emerge. The more precocious manifestation of AML is most likely the result of the presence of AML-propagating cells in the transplant carrying additional disease-driving genetic lesions that may accelerate tumor growth. Alternatively, the WT marrow of the transplantation model could present a more conducive microenvironment for malignant transformation than the *Crebbp*^*+/-*^ microenvironment.

Unfractionated BM comprises a complex mixture of cell types that can contribute separately or in concert to disease development. For example, transplantation of unfractionated *lyn*^-/-^*PLC-β3*^-/-^ BM resulted in MDS/MPN, whereas purified *lyn*^-/-^*PLC-*β*3*^-/-^ long-term repopulating HSCs gave rise to an isolated MPN [46]. In our case, half the animals receiving unfractionated *Crebbp*^*+/-*^ BM developed early-onset AML but none of those receiving purified LSKs did (Fig 1). Nor was there evidence of the myeloproliferation previously observed in naïve *Crebbp*^*+/-*^ animals and in WT BM cells exposed to the *Crebbp*^*+/-*^ microenvironment [2, 16]. Rather, LSK recipients overwhelmingly developed a tri-lineage MDS after 11–17 months. It would therefore appear that MDS- and AML-propagating cells reside in different fractions of the *Crebbp*^*+/-*^ BM, though the initiating cells may or may not be distinct. It is possible that the immunophenotype of LSK cells was altered by leukemic transformation, as has been documented in CALM/AF10-positive AML [47]; if this were the case, the isolation approach we used might have excluded AML-propagating cells. An alternative explanation is that AML development by LSKs in this model requires additional signals from other *Crebbp*^*+/-*^ BM subpopulations. In particular, the fact that *Crebbp*^*+/-*^ CMPs or GMPs triggered myeloproliferation in recipients' WT cells could tie in with the requirement for AML-initiating cells to receive differentiation signals in order to elicit their full disease potential [48]. If the WT microenvironment did not provide those signals, then the *Crebbp*^*+/-*^ LSKs may have "stalled" before giving rise to a discernable leukemia.

Our findings in mice transplanted with *Crebbp*^*+/-*^ CMPs or GMPs also support a model for MDS/MPN-U where distinct cellular components of the BM and its microenvironment contribute to disease development. In 4 of 24 animals, more than 10% of the cells of a specific lineage were dysplastic, sufficient to warrant a diagnosis of MDS. Of particular importance, the hematologic aberrations observed in recipients of *Crebbp*^*+/-*^ CMPs or GMPs originated from WT cells rather than from transplanted *Crebbp*^*+/-*^ cells or their progeny. We showed that *Crebbp*^+/-^ mature progeny release an abundance of MMP9 and display an altered cytokine, exosomal and extracellular matrix gene expression profile (Fig 6). This particular profile of alterations in mature *Crebbp*^+/-^ myeloid cells is distinct from any previously reported TMC population and supports the notion that each tumor may be associated with a unique myeloid cell population [49]. The increased MMP9 production is of particular interest because the role of MMP9 in promoting tumor growth is well-established [30]. Therefore, our data are consistent with the hypothesis that the transplanted *Crebbp*^*+/-*^ CMPs and GMPs may induce persistent changes in the recipients' hematopoietic system through the aberrant actions of their myeloid progeny. Intriguingly, non-hematopoietic stromal cells of a *Crebbp*^*+/-*^ genotype show a decrease in MMP9 [16], which was interpreted as one of the reasons why the *Crebbp*^*+/-*^ microenvironment is incapable of maintaining HSCs [16]. These studies emphasize the effect of the cellular context when studying the biological consequences of *Crebbp* hemizygosity on the functionality of the BM microenvironment.

The reprogramming of normal function by cells harboring mutations has been documented in other murine models of human disorders. For example, in immunocompromised mice transplanted with human cord blood cells expressing TEL-JAK2, myelofibrosis rapidly developed but the clusters of atypical megakaryocytes with hyperlobulated nuclei were found to be of murine in origin [50]. Similarly, osteogenic loss of *Dicer* caused pancytopenia and megakaryocytic and granulocytic dysplasia in genotypically WT hematopoietic cells and these abnormalities were also apparent when WT BM cells were transplanted into *Dicer*-deficient recipients [51]. The ability of mutation-bearing disease cells to alter the phenotype of WT cells observed in mice may explain some of the puzzling observations made in secondary AML that occurs in a subset of patients with an MPN [52–54]. Here, it was found that the mutant JAK2 allele is not present in the leukemic blast cells of half of all JAK2V617F-positive MPN patients who develop AML [55, 56]. Molecular analysis of paired MPN and AML samples from several of these cases showed that this was not due to deletion of the mutant JAK2 locus or loss of chromosome 9, or to homologous recombination within a JAK2V617F-heterozygous cell [57]. Instead, the JAK2-mutated MPN clone and JAK2-wildtype leukemic clone were found to have distinct mutation profiles; for example, an acquired del(20q) was present in the JAK2-mutant erythroid progenitor cells of one patient, but not in his purified leukemic blasts. This finding suggests that the two disorders arise in independent HSCs. It is possible that these disorders share a haplotype that predisposes them to acquiring mutations; alternatively, a JAK2-mutant HSCs, or its more mature daughter cells, may induce genetic changes in other (JAK2-wildtype) HSCs.

One can thus imagine that MDS and AML in *Crebbp*^*+/-*^ animals (and potentially secondary AML in MPN) results from the coordinated, aberrant action of several marrow components—not necessarily all bearing mutations—rather than from the isolated activity of a unique, phenotypically defined disease progenitor. Such a situation would present quite different treatment challenges than the more common approach of targeting a specific mutation in a defined cell population.

## Supporting Information

S1 Fig Experimental design and reconstitution outcome of the transplantation studies. (PDF)

S2 Fig Leukemia in the recipients transplanted with unfractionated *Crebbp*^+/-^ BM cells originate from the myeloid lineage. (PDF)

S3 Fig Leukemic progression of MDS in a recipient of unfractionated *Crebbp*^+/-^ BM. (PDF)

S4 Fig Increased myelopoiesis in recipients of unfractionated *Crebbp*^+/-^ BM who did not develop early-onset AML. (PDF)

S5 Fig Transplant recipient of *Crebbp*^+/-^ LSKs develop late-onset acute T-cell leukemia and chronic myelomonocytic leukemia. (PDF)

S6 Fig Methodology to determine the presence of CD45.2^+^ LSK and LS^-^K cells in CD45.1^+^ recipients of Crebbp^+/-^;CD45.2^+^ CMPs or GMPs. (PDF)

S7 Fig No evidence for abnormal myelopoiesis in WT recipients transplanted with low numbers of WT, unfractionated BM cells. (PDF)

S1 Table Experimental details of *Crebbp*^*+/-*^ BM transplantation studies. (PDF)

S2 Table Comparison of the number of cells received from inoculums of unfractionated cells and purified cells. (PDF)

S3 Table CD45.2-derived reconstitution and disease outcome in wild-type recipients following transplantation of *Crebbp*^*+/-*^ CMPs and GMPs. (PDF)

S4 Table Peripheral blood parameters in wild-type recipients of *Crebbp*^*+/-*^ CMPs and GMPs. (PDF)

S5 Table Raw and normalized data of the NanoString gene expression analysis. (XLS)
