# Supplementary material for: Context Matters: Distinct Disease Outcomes as a Result of Crebbp Hemizygosity in Different Mouse Bone Marrow Compartments
Source: PLoS One. 2016 Jul 18;11(7):e0158649. doi: 10.1371/journal.pone.0158649 (PMC4948888; doi:10.1371/journal.pone.0158649)
Supplement: S1 Fig — (PDF) [file pone.0158649.s001.pdf]

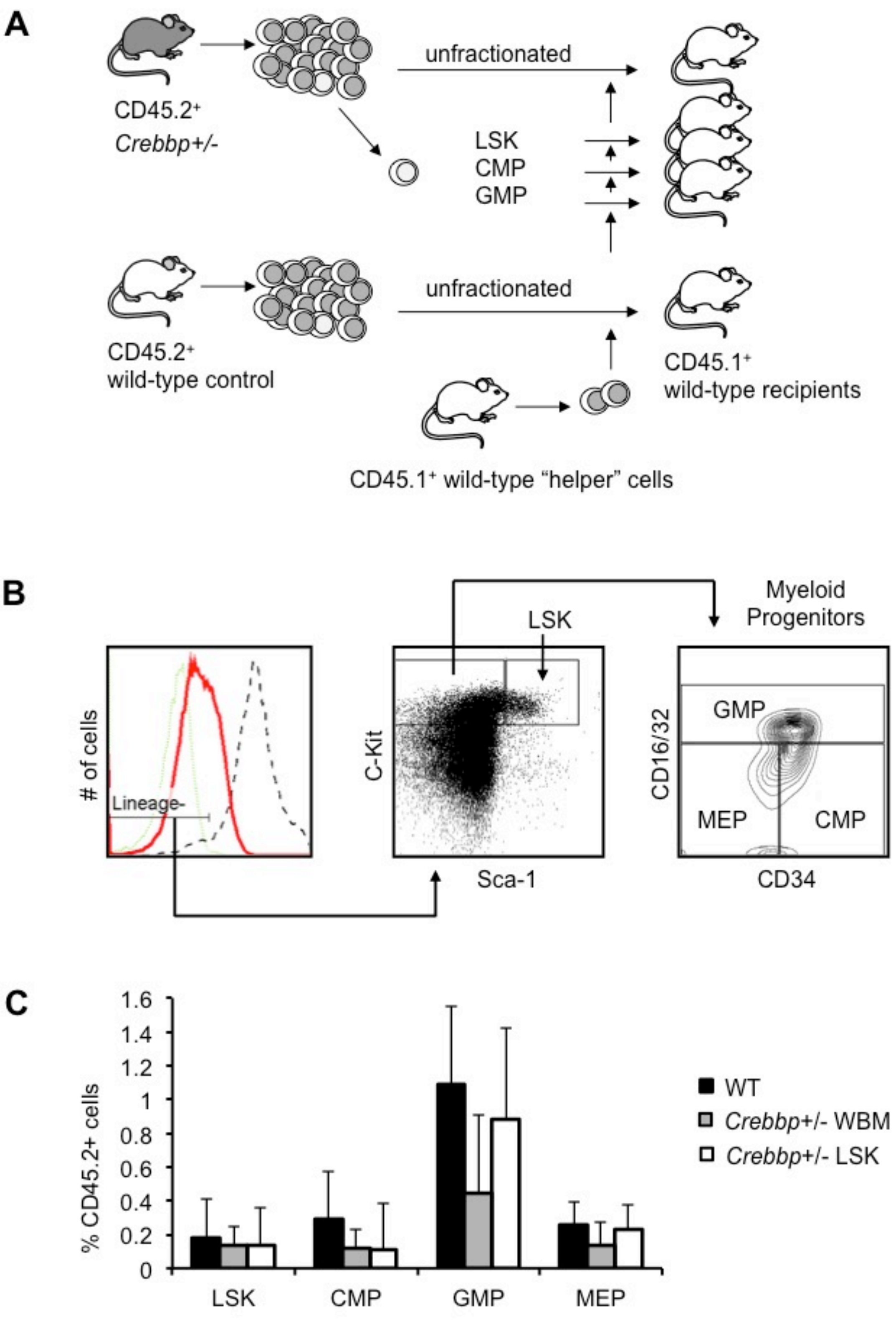
A
CD45.2+
Crebbp+/-
unfractionated
LSK
CMP
GMP
CD45.2+
wild-type control
unfractionated
CD45.1+
wild-type recipients
CD45.1+ wild-type "helper" cells
B
# of cells
Lineage-
C-Kit
Sca-1
LSK
Myeloid
Progenitors
CD16/32
GMP
MEP
CMP
CD34
C
% CD45.2+ cells
1.6
1.4
1.2
1
0.8
0.6
0.4
0.2
0
LSK
CMP
GMP
MEP
WT
Crebbp+/- WBM
Crebbp+/- LSK

S1 Fig.

S1 Fig. Experimental design and reconstitution outcome of the transplantation studies. (**A,** transplantation set-up) Unfractionated bone marrow (BM) cells or FACS-purified LSKs, CMPs and GMPs from *Crebbp*$^{+/-}$ donors were transplanted into separate groups of irradiated wild-type recipients (n=3 or more per group per experiment). All recipients also received 2 x 10^5 wild-type BM cells to ensure survival ("helper" cells). Equal numbers of age-matched unfractionated wild-type BM were transplanted to serve as controls. (**B,** sorting strategy) Lineage depleted (Lin$^-$) BM cells (left panel) are investigated for LSKs and myeloid progenitors. The black dashed line indicates the lineage marker staining in unfractionated BM, while the green dashed line indicates the IgG isotype control. The gate for Lin$^-$ BM cells lies where these two histograms overlap. The red line indicates the lineage marker staining in BM cells after lineage-depletion. Within the Lin$^-$ BM cells, LSKs express high levels of c-Kit and Sca-1, while myeloid progenitors express high levels of c-Kit but are negative for Sca-1 (middle panel). The myeloid progenitors were further investigated for expression of CD34 and CD16/32, which identifies subsets of committed myeloid progenitors, as indicated by the boxes (right panel). (**C,** reconstitution outcome) Depicted are the average percentages + SD of donor-derived (CD45.2$^+$) LSKs, CMPs, GMPs and MEPs, measured in recipients of unfractionated wild-type BM (black bars), unfractionated *Crebbp*$^{+/-}$ BM (grey) and *Crebbp*$^{+/-}$ LSKs (white) at the time of sacrifice. A similar gating strategy was used as presented in (B) to identify LSKs, CMPs and GMPs; MEPs are cells with a Lin$^-$Sca-1$^-$ ckit$^+$CD34$^-$CD16/32$^-$ phenotype. These results show that the reconstitution of immature hematopoietic subsets does not differ between the various groups of transplant recipients tested.
