# Supplementary material for: Context Matters: Distinct Disease Outcomes as a Result of Crebbp Hemizygosity in Different Mouse Bone Marrow Compartments
Source: PLoS One. 2016 Jul 18;11(7):e0158649. doi: 10.1371/journal.pone.0158649 (PMC4948888; doi:10.1371/journal.pone.0158649)
Supplement: S2 Fig — (PDF) [file pone.0158649.s002.pdf]

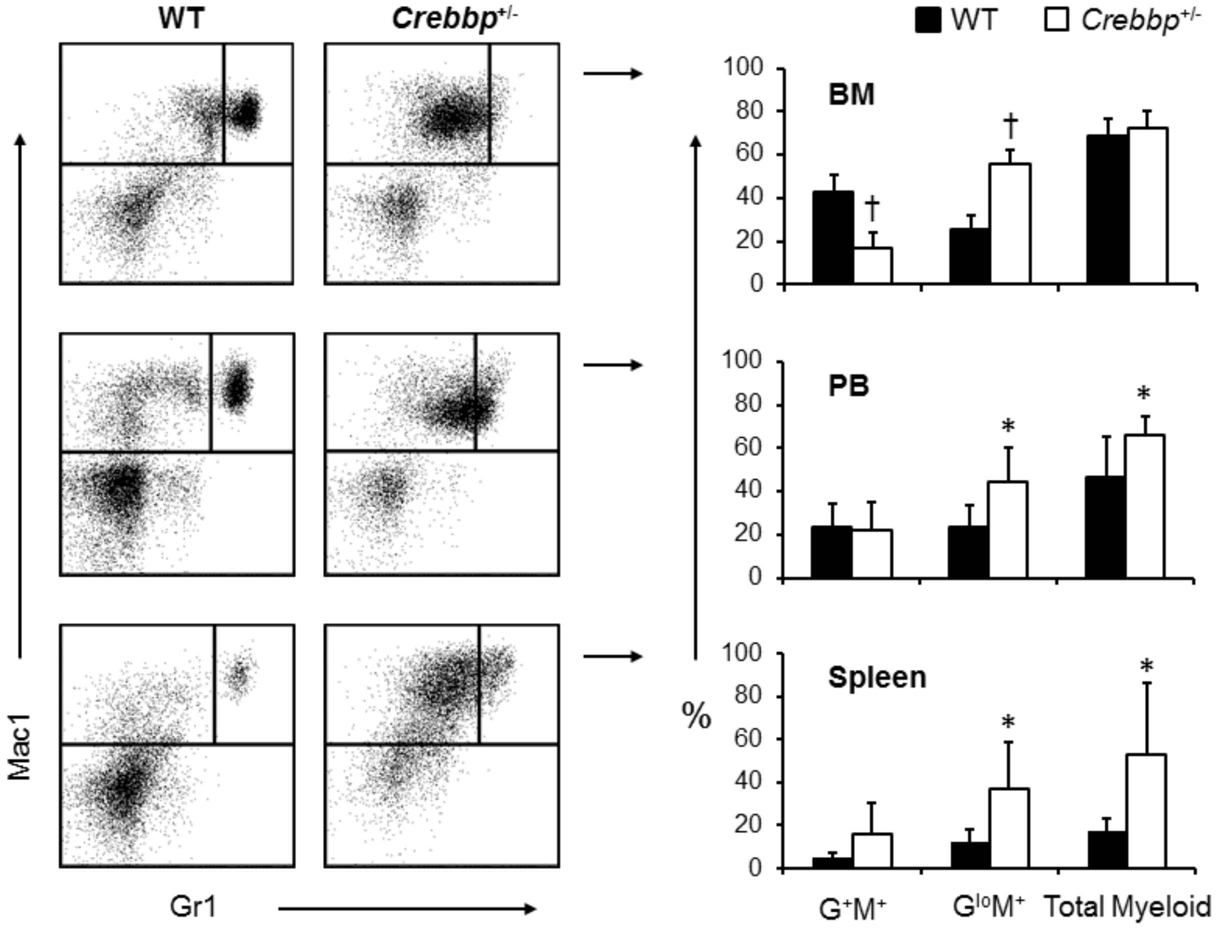

S2 Fig. Leukemia in recipients transplanted with unfractionated *Crebbp*$^{+/-}$ BM cells originate from the myeloid lineage. Representative FACS profiles of CD45.2^{+} donor-derived cells in BM cells (upper panel of figures), PB (middle) and Spleen (bottom) from transplant recipients of wild-type (black bars, n=10) and *Crebbp*$^{+/-}$ BM cells (white bars, n=8). The latter were all diagnosed with AML. The bar graphs present the average percent + SD of Gr1^{lo}Mac1^{+} (G^{lo}M^{+}) cells (upper left gate in profile), Gr1^{+}Mac1^{+} (G^{+}M^{+}) cells (upper right gate in profile) and total myeloid cells (sum of the former two populations) of donor-derived cells in the same two groups of mice. Significant differences between wild-type and *Crebbp*$^{+/-}$ transplant recipients are indicated by the following symbols: * $p<0.05$, † $p< 0.001$.
