# Supplementary material for: Context Matters: Distinct Disease Outcomes as a Result of Crebbp Hemizygosity in Different Mouse Bone Marrow Compartments
Source: PLoS One. 2016 Jul 18;11(7):e0158649. doi: 10.1371/journal.pone.0158649 (PMC4948888; doi:10.1371/journal.pone.0158649)
Supplement: S3 Fig — (PDF) [file pone.0158649.s003.pdf]

A i

WBC (X10⁹/L)	RBC (X10¹²/L)	PLT (X10⁹/L)	Spleen weight (g)
28.84	7.86	708	0.3

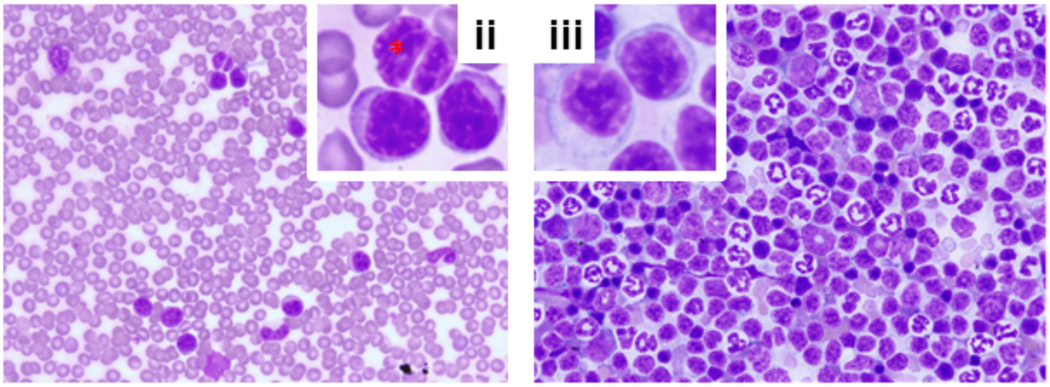

B

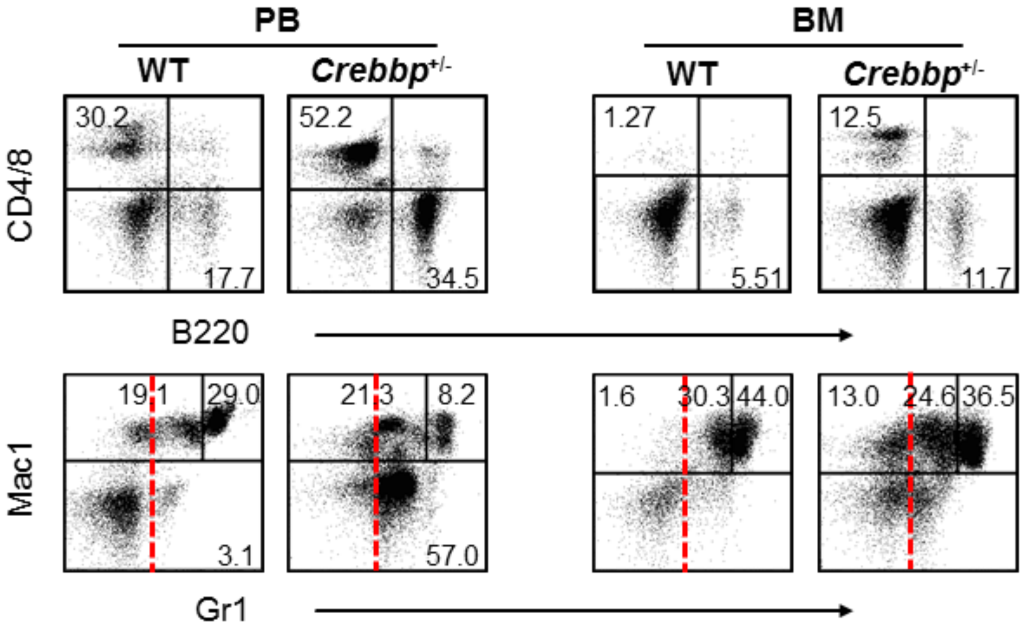

C

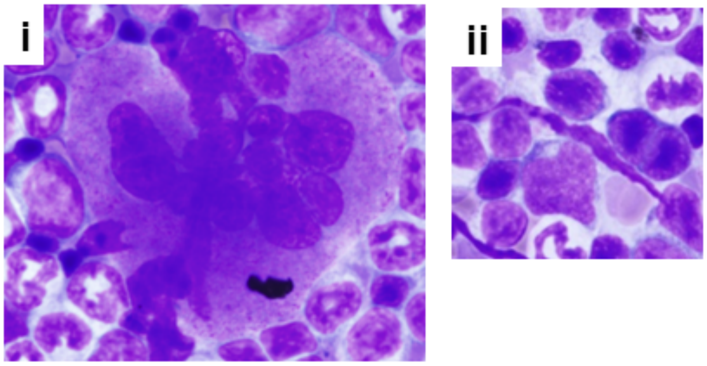

S3 Fig.

S3 Fig. Leukemic progression of MDS in a recipient of unfractionated *Crebbp*$^{+/-}$ BM. (**A**) (**i**) WBCs, RBCs, PLTs and spleen weights. (**ii**) Giemsa-stained blood smear showing leukocytosis; the insert shows the co-existence of a pseudo Pelger-Huet anomaly (indicated by the red star) and cells with lymphocytic morphology, which represents the majority of the cells in the PB. (**iii**) BM touch preparation showing the dominance of cells with lymphocytic morphology (shown at higher magnification in inset), at the cost of the erythroid lineage. (**B**) FACS profiles of PB cells (left panel) and BM cells (right panel) from a representative control receiving wild-type cells and the recipient of interest that received *Crebbp*$^{+/-}$ cells. Single cell suspensions were stained with antibodies directed against mature lymphoid cells markers (B220 for B cells and CD4 and CD8 for T cells) or mature myeloid markers (Gr1 and Mac1). The numbers indicate the proportion of CD45.2^{+} donor-derived cells with the phenotype corresponding to the respective gate. The red line in each of the lower FACS panels separates cells that do not express Gr1 (left) from those that do (right). The majority of the PB cells in the *Crebbp*$^{+/-}$ transplant recipient express a B or a T cell marker, which corresponds well with the histological PB observations (**Ai**), however, flow cytometric analysis demonstrates that most PB cells also express low levels of the myeloid marker Gr1. In the BM, there is an increase of cells with mature lymphoid markers compared to wild-type. The distribution of myeloid markers on the cells of the BM cells is changed as well. (**C**) Giemsa-stained BM cells touch preparations showing myelodysplastic features in the form of multi-nucleated megakaryocytes (**i**) and binucleated erythroid precursors (**ii**), suggesting that the leukemic disease was likely preceded by MDS. Magnification: ×40 (**A**) and ×60 (**C**).
