# Supplementary material for: Context Matters: Distinct Disease Outcomes as a Result of Crebbp Hemizygosity in Different Mouse Bone Marrow Compartments
Source: PLoS One. 2016 Jul 18;11(7):e0158649. doi: 10.1371/journal.pone.0158649 (PMC4948888; doi:10.1371/journal.pone.0158649)
Supplement: S4 Fig — (PDF) [file pone.0158649.s004.pdf]

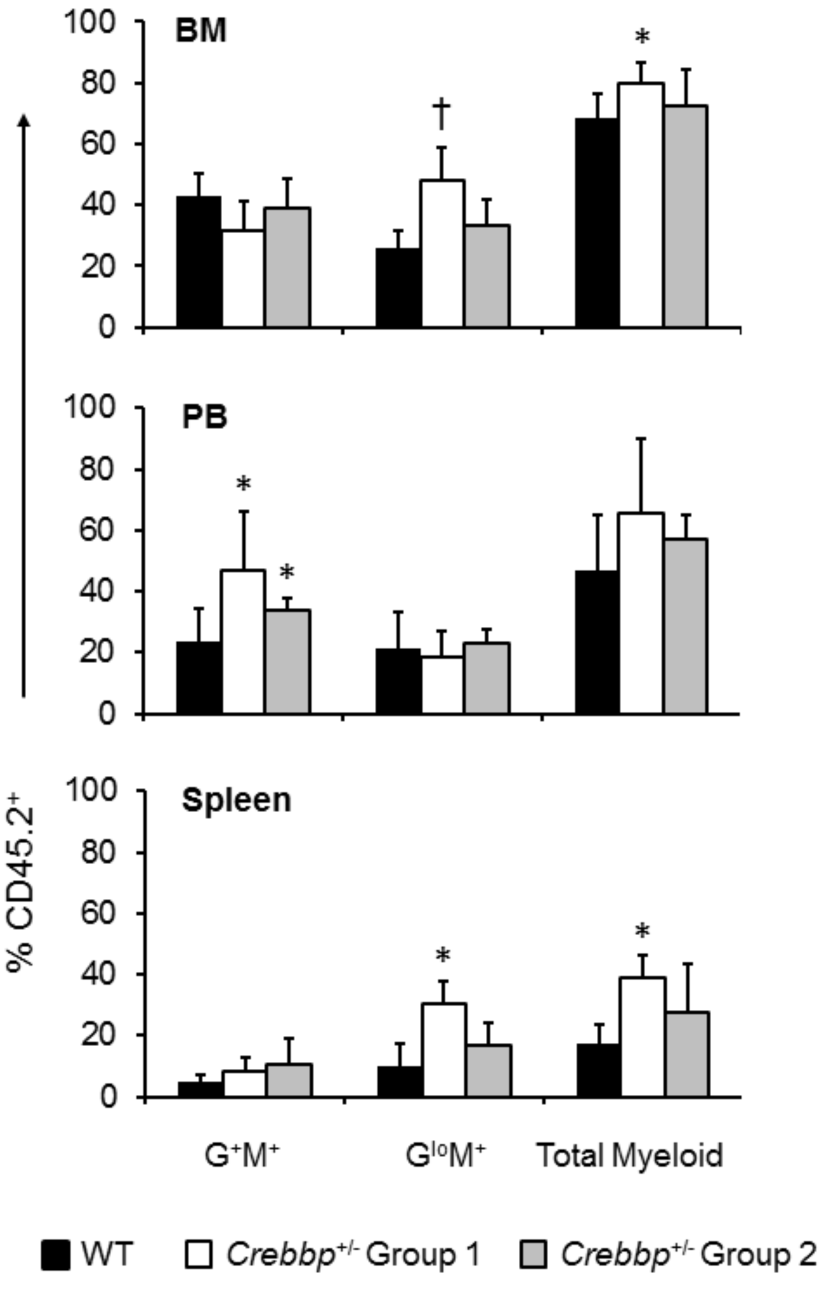

S4 Fig. Increased myelopoiesis in recipients of unfractionated *Crebbp*$^{+/-}$ BM who did not develop early-onset AML. Presented are the average percent + SD of Gr1^{+}Mac1^{+} (G^{+}M^{+}), Gr1^{lo}Mac1^{+} (G^{lo}M^{+}) and total myeloid cells in CD45.2^{+}, donor-derived cells in the BM (upper), PB (middle) and spleen (bottom) of wild-type controls (black bars; n=10) and recipients of unfractionated *Crebbp*$^{+/-}$ BM cells. The latter group was divided in two subgroups based on the absence (group 1, white bars, n=5) or presence of thrombocytosis (group 2, gray bars, n=4).
* $p<0.05$, † $p< 0.001$.
