# Supplementary material for: Context Matters: Distinct Disease Outcomes as a Result of Crebbp Hemizygosity in Different Mouse Bone Marrow Compartments
Source: PLoS One. 2016 Jul 18;11(7):e0158649. doi: 10.1371/journal.pone.0158649 (PMC4948888; doi:10.1371/journal.pone.0158649)
Supplement: S5 Fig — (PDF) [file pone.0158649.s005.pdf]

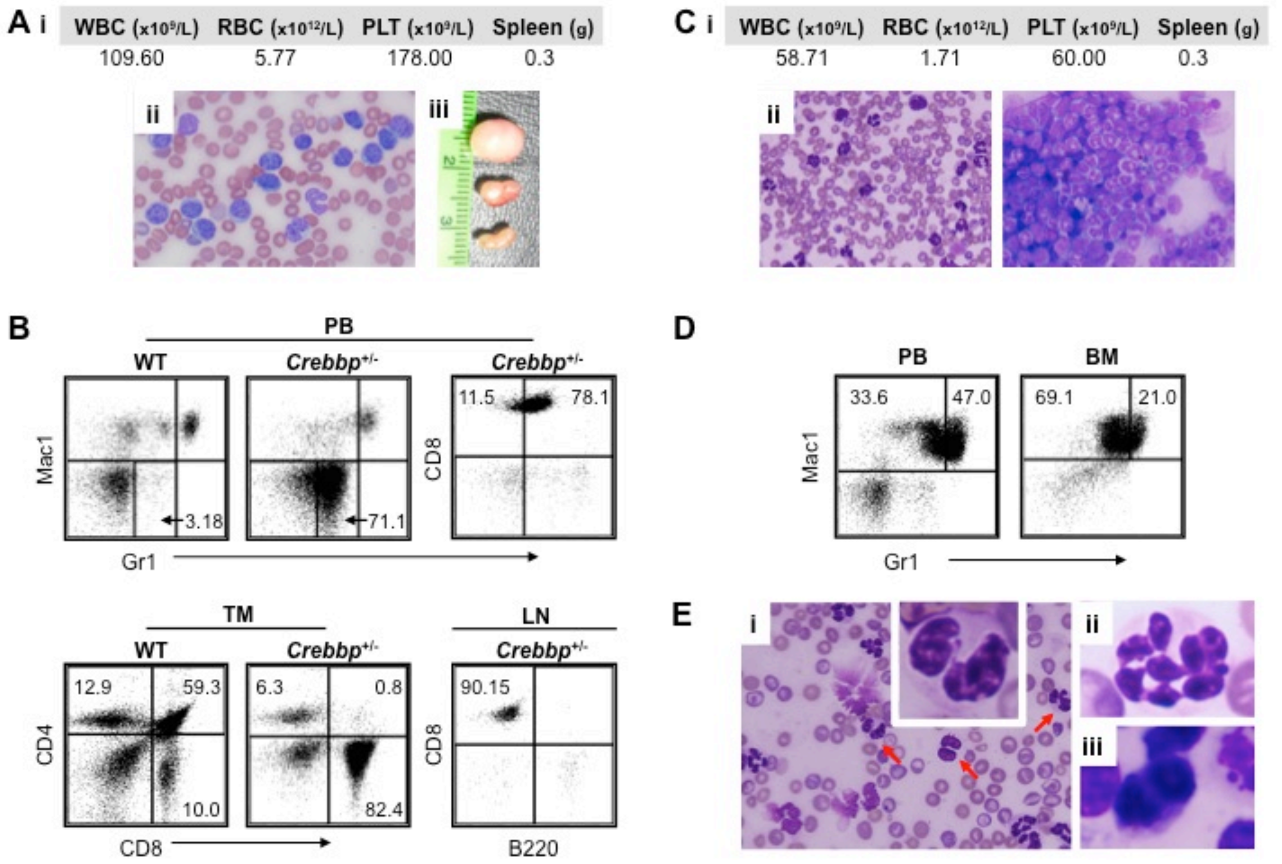

S5 Fig. Transplant recipients of *Crebbp*$^{+/-}$ LSKs develop late-onset acute T-cell leukemia and chronic myelomonocytic leukemia. (A, B) Late-onset acute T-cell leukemia (**A**) (**i**) WBCs, RBCs, PLTs and spleen weights. (**ii**) Giemsa-stained PB smear showing leukocytosis due to accumulation of morphologically mature lymphocytes. (**iii**) Enlarged lymph nodes. Magnification: ×40. (**B**) FACS profiles of PB, thymus (TM) and lymph node cells (LN) stained with antibodies directed against myeloid markers (Mac1 and Gr1) and lymphoid markers (B220, CD4 and CD8). Numbers indicate the proportion of corresponding cell populations of total CD45.2^{+} donor-derived cells. Compared to the WT control (left), the recipient of *Crebbp*$^{+/-}$ LSKs showed a remarkably increased Gr1^{lo}Mac1^{-} subpopulation in the PB that also expressed the CD8 T-cell marker. Consistent with the PB findings, the TM from this recipient showed a significant increase in CD4^{-}CD8^{+} thymocytes and the large LN was comprised of mostly CD8^{+} cells.

(C-E) Chronic myelomonocytic leukemia. (**C**) (**i**) WBCs, RBCs, PLTs and spleen weights. (**ii**) Giemsa-stained blood smear (left) showing significant leukocytosis with predominance of well-differentiated granulocytes and a significant increase in monocytes. Giemsa-stained BM touch preparation (right) showing <20% myeloblasts; predominant cell type in the BM are mature granulocytes. (**D**) Representative FACS profiles of CD45.2^{+}, donor-derived PB (left panel) and BM (right panel). Cells were stained with antibodies

directed against the myeloid markers Mac1 and Gr1. Numbers indicate the proportion of corresponding myeloid cell populations of total $CD45.2^+$ donor-derived cells. (**E**) Giemsa-stained PB smears (**i-ii**) and a BM touch preparation (**iii**) showing myelodysplasia evidenced by pseudo Pelger-Huet anomalies (**i**,arrows and inset), hypersegmented granulocytes (**ii**) and binucleated erythroid precursors (**iii**). Magnification: ×40 (**Aii-iii**) and ×60 (**Bi-iii**).
