# Supplementary material for: Context Matters: Distinct Disease Outcomes as a Result of Crebbp Hemizygosity in Different Mouse Bone Marrow Compartments
Source: PLoS One. 2016 Jul 18;11(7):e0158649. doi: 10.1371/journal.pone.0158649 (PMC4948888; doi:10.1371/journal.pone.0158649)
Supplement: S6 Fig — (PDF) [file pone.0158649.s006.pdf]

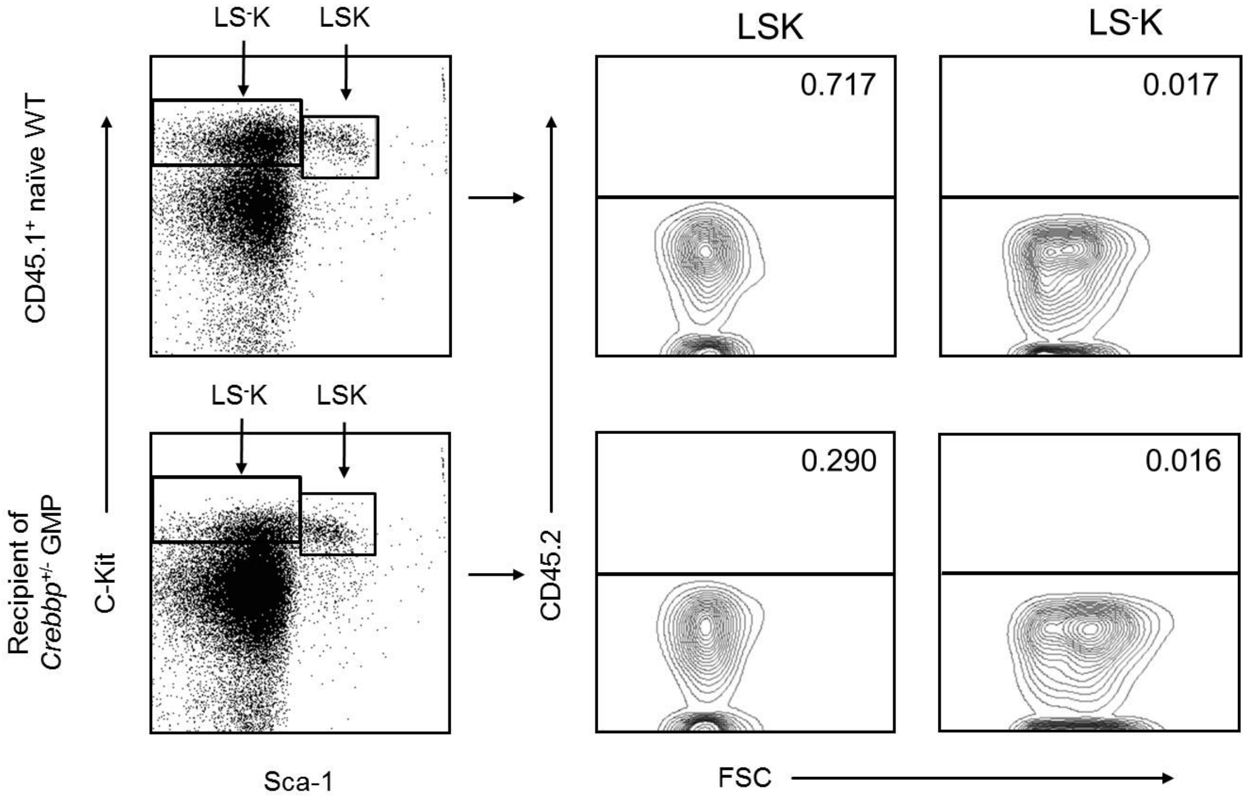

S6 Fig. Methodology to determine the presence of CD45.2$^+$ LSK and LS$^-$K cells in CD45.1$^+$ recipients of *Crebbp*$^{+/-}$;CD45.2$^+$ CMPs or GMPs. Representative FACS profiles are shown for a naïve CD45.1$^+$ mouse (top three profiles) and a CD45.1$^+$ mice transplanted with CD45.2$^+$ GMPs (bottom three profiles). The mice were age and sex-matched. Bone marrow was harvested as usual and stained with a cocktail of antibodies directed at mature lineage markers, Sca-1, c-Kit and CD45.2, and a marker to exclude dead cells. The far left profiles show live cells that lack expression of mature lineage markers. The LSK gate indicates the stem/progenitor population and the LS$^-$K gate the myeloid progenitor population. Each of these populations was then further analyzed for CD45.2 expression (middle and far right profiles). The analysis of naïve mice (n=7) served to determine background levels of CD45+ staining in each population. On average, 1.42 (± 1.69) % of LSK cells in naïve CD45.1$^+$ mice stained positive for CD45, while this percentage was 0.27 (± 0.32) % for the LS$^-$K myeloid progenitor population. A proportion of CD45.2$^+$ LSKs or LS$^-$Ks in CMP or GMP transplant recipient was considered significant if it exceeded the average + 2 SD in the naïve CD45.1 controls, i.e., 4.8% and 0.9% for LSK and LS$^-$K cells, respectively.
