# Supplementary material for: Context Matters: Distinct Disease Outcomes as a Result of Crebbp Hemizygosity in Different Mouse Bone Marrow Compartments
Source: PLoS One. 2016 Jul 18;11(7):e0158649. doi: 10.1371/journal.pone.0158649 (PMC4948888; doi:10.1371/journal.pone.0158649)
Supplement: S7 Fig — (PDF) [file pone.0158649.s007.pdf]

A

	WBC ($X10^9/L$)	RBC ($X10^{12}/L$)	PLT ($X10^9/L$)	Spleen Weight (g)
2×10^5	$4.95 \pm 2.01^{\dagger}$	$7.87 \pm 0.72^{*}$	652.11 ± 200.38	0.09 ± 0.03
5×10^6	10.09 ± 3.25	9.15 ± 0.84	485.14 ± 135.15	0.10 ± 0.00

B

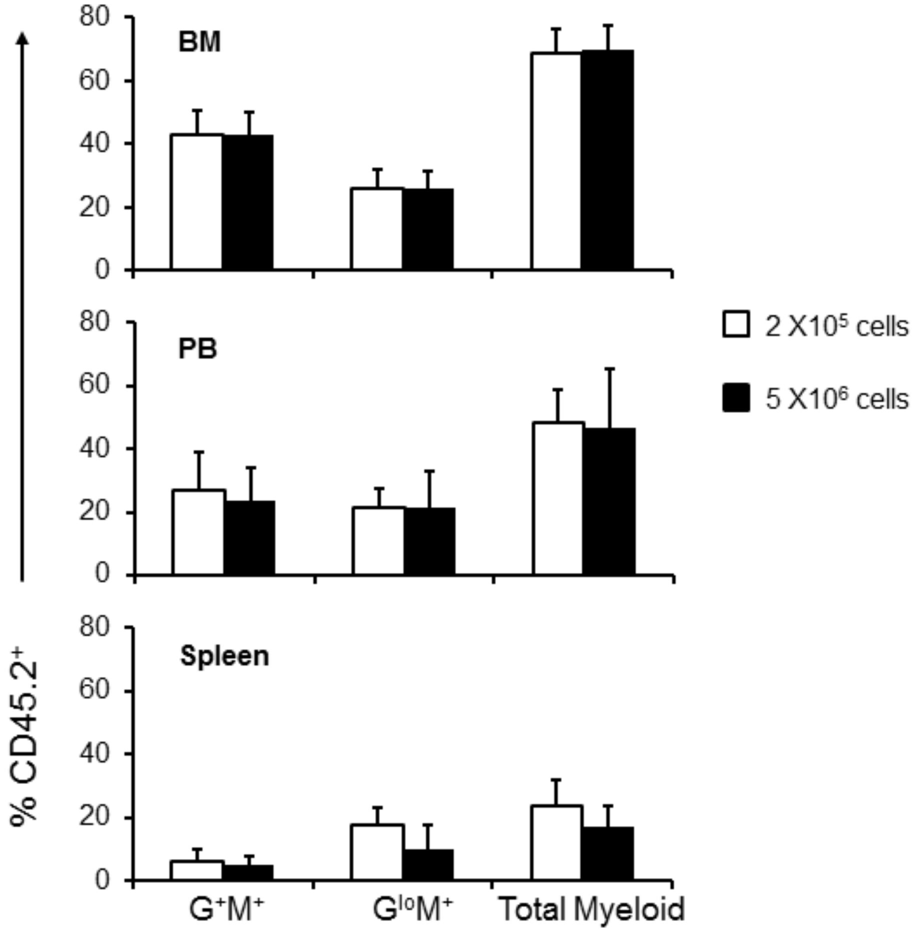

C

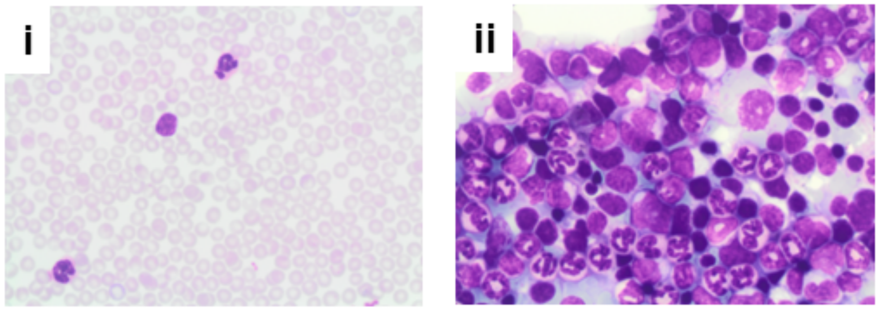

S7 Fig.

S7 Fig. No evidence for abnormal myelopoiesis in WT recipients transplanted with low numbers of WT, unfractionated BM cells. Although hematopoietic reconstitution seems less robust in recipients transplanted with 2 x 10^5 cells compared to recipients that received 5 x 10^6 cells (**A**), there is no evidence of a relative increase in myelopoiesis (**B**), myelodysplasia or other hematopoietic disease (**C**). Measurements were made 15 months post-transplantation. (**A**) Average ± SD of WBCs, RBCs, PLTs and spleen weights after receiving 2 x 10^5 cells and 5 x 10^6 cells. * p=0.022, † p=0.002. (**B**) Data present average percent + SD of donor-derived $Gr1^+Mac1^+$ (G^+M^+), $Gr1^{lo}Mac1^+$ ($G^{lo}M^+$) and total myeloid cells in the BM (upper panel), PB (middle) and spleen (bottom) of wild-type recipients transplanted with 2 x 10^5 (white bars, n=12) and 5 x 10^6 (black bars; n=10) WT, unfractionated BM cells. No significant difference was observed between two groups. (**C**) Representative images of PB smear (**i**) and BM touch preparation (**ii**) from recipients that received 2 x 10^5 cells showing normal histology. Magnification: ×40.
