# Supplementary material for: Context Matters: Distinct Disease Outcomes as a Result of Crebbp Hemizygosity in Different Mouse Bone Marrow Compartments
Source: PLoS One. 2016 Jul 18;11(7):e0158649. doi: 10.1371/journal.pone.0158649 (PMC4948888; doi:10.1371/journal.pone.0158649)
Supplement: S1 Table — (PDF) [file pone.0158649.s008.pdf]

S1 Table. Experimental details of *Crebbp*$^{+/-}$ BM transplantation studies.

Experiment	Transplanted cell type * (# of recipients)		Outcome (# of recipients)		Sacrifice †	Disease genotype
1	UBM	(3)	AML	(3)	6.2	*Crebbp*$^{+/-}$
	LSK	(3)	MDS	(2)	13.3	*Crebbp*$^{+/-}$
			T-cell leukemia	(1)	13.8	
	CMP	(2)	No disease	(2)	13.8	
	GMP	(3)	No disease	(3)	12.9	
2	UBM	(8)	AML	(1)	4.9	*Crebbp*$^{+/-}$
			MDS	(5)	12.9	
			MDS/MPN	(2)	17.3	
	LSK	(10)	MDS	(9)	14.4	*Crebbp*$^{+/-}$
			CMML	(1)	13.9	
	CMP	(8)	MDS	(2)	13.6	WT
			AML	(1)	11.6	
			No disease	(5)	14.3	
	GMP	(11)	MDS	(2)	13.9	WT
			MPN	(2)	13.7	
			No disease	(7)	14.7	
3	UBM	(4)	AML	(4)	3.7	*Crebbp*$^{+/-}$
	LSK	(4)	MDS	(4)	11.0	*Crebbp*$^{+/-}$
4	UBM	(3)	MDS/MPN	(2)	14.8	*Crebbp*$^{+/-}$
			MDS/MPN with leukemic transformation	(1)	16.4	

* All cells for transplantation were obtained from *Crebbp*$^{+/-}$ donors
† Expressed as average number of months post-transplantation
UBM = unfractionated bone marrow

The characteristics of AML, MDS and MDS/MPN did not differ from one experiment to the other and we therefore decided to pool the data. Individual experiments included 4-5 controls that received WT UBM (not shown).
