# Supplementary material for: Context Matters: Distinct Disease Outcomes as a Result of Crebbp Hemizygosity in Different Mouse Bone Marrow Compartments
Source: PLoS One. 2016 Jul 18;11(7):e0158649. doi: 10.1371/journal.pone.0158649 (PMC4948888; doi:10.1371/journal.pone.0158649)
Supplement: S2 Table — (PDF) [file pone.0158649.s009.pdf]

S2 Table. Comparison of the number of cells received from inoculums of unfractionated cells and purified cells.

	Unfractionated BM	Purified populations
**LSK**	10,393 - 23,221	10,000 - 25,000
**CMPs**	12,498 - 17,458	10,000 - 25,000
**GMPs**	21,096 - 38,297	10,000 - 50,000

The numbers of respective populations contained in the unfractionated BM = the frequency of each population in BM (determined by FACS analysis) x number of unfractionated BM cells transplanted. For example, in BMT1, 4.35 x 10^6 unfractionated BM was transplanted into each recipient and the frequency of LSKs in that BM cell suspension was 0.24%. Therefore, the number of LSKs contained in each inoculum of unfractionated BM = 4.35 x 10^6 x 0.24% = 10,393.
