# Supplementary material for: Context Matters: Distinct Disease Outcomes as a Result of Crebbp Hemizygosity in Different Mouse Bone Marrow Compartments
Source: PLoS One. 2016 Jul 18;11(7):e0158649. doi: 10.1371/journal.pone.0158649 (PMC4948888; doi:10.1371/journal.pone.0158649)
Supplement: S3 Table — (PDF) [file pone.0158649.s010.pdf]

S3 Table. CD45.2-derived reconstitution and disease outcome in WT recipients following transplantation of *Crebbp*$^{+/-}$ CMPs and GMPs.

Mouse ID	Sacrifice *	% CD45.2^{+} cells †				BM Dysplasia	Splenomegaly	Diagnosis ≠
		PB	BM	LSK	LS^{-}K			
**CMP recipients**								
1	15.0	10.0	2.8	0.7	0.8	E, MK, M < 10%	No	
2	12.7	0.6	1.6	0.3	0.2	No	No	
3	14.2	0.1	0.1	0.0	0.0	E, MK, M > 10%	No	MDS
4	13.0	0.1	0.7	0.2	0.1	E, MK, M > 10%	No	MDS
5	11.6	0.3	0.6	ND	ND	No §	Yes (0.30 g)	AML, Myelofibrosis
6	15.9	0.4	0.3	0.0	0.0	No	No	
7	17.4	0.2	0.0	0.6	0.2	No	No	
8	17.5	0.8	0.1	0.9	0.2	MK < 10%	No	
9	10.4	0.1	0.1	1.2	0.9	M < 10%	Yes (0.25 g)	
10	10.4	0.2	0.1	1.9	2.7	No	Yes (0.25 g)	
**GMP recipients**								
11	12.0	4.9	ND	ND	ND	E: anisocytosis	No	
12	14.5	2.2	2.4	0.1	0.1	E < 10%	No	
13	12.2	7.7	3.7	1.4	2.0	M < 10%	No	
14	10.4	0.3	0.1	0.6	0.2	No	No	
15	14.6	0.3	0.0	1.3	0.1	No	No	
16	10.4	0.1	0.2	0.2	0.2	No	No	MPN
17	16.3	0.8	0.1	0.2	0.3	E, MK, M < 10%	No	
18	13.6	0.2	0.1	1.0	0.1	E < 10%	No	
19	14.2	0.1	0.1	0.1	0.1	E > 10%	No	MDS
20	13.5	0.1	0.2	0.2	0.0	M, E > 10%	Yes (0.25 g)	MDS
21	15.9	0.5	0.0	0.0	0.0	No	Yes (0.25 g)	
22	18.9	1.0	0.5	0.0	0.0	E, MK, M < 10%	No	
23	13.0	0.0	0.0	0.3	0.2	E, MK, M < 10%	Yes (0.40 g)	
24	16.9	0.1	0.5	0.7	0.4	No	No	MPN

Mice 1 and 2 received 25,000 CMPs, mice 3-10 received 10,000 CMPs.
Mice 11-13 received 50,000 GMPs, mice 14-18 received 25,000 GMPs and mice 19-24 received 10,000 GMPs.
LS^{-}K = Lin^{-}Sca-1^{-}cKit^{++} cells; E = erythroid cells; MK = megakaryocytes; M = myeloid cells.
* Months post-transplantation.
† CD45.2^{+} cells are of *Crebbp*$^{+/-}$ genotype. Provided is the percentage CD45.2^{+} reconstitution at time of sacrifice. No significant CD45.2^{+} reconstitution was observed in the LSK population and only 2 mice (#10 and #13) were considered to have significant CD45.2^{+} reconstitution in the LS^{-}K population. Please see **S8 Fig** for an explanation on how these numbers were obtained for the LSK and LS^{-}K populations in the BM.

≠ Additional hematological parameters required to make a diagnosis are provided for each animal in **S4 Table**.

§ Dysplasia was absent, but the marrow was hypocellular (2.88 X 10^6 cells per 2 femurs) and showed > 20% blasts and myelofibrosis (**Fig 4C**).
