# Supplementary material for: Context Matters: Distinct Disease Outcomes as a Result of Crebbp Hemizygosity in Different Mouse Bone Marrow Compartments
Source: PLoS One. 2016 Jul 18;11(7):e0158649. doi: 10.1371/journal.pone.0158649 (PMC4948888; doi:10.1371/journal.pone.0158649)
Supplement: S4 Table — (PDF) [file pone.0158649.s011.pdf]

Table S4. PB parameters in WT recipients of *Crebbp*$^{+/-}$ CMPs and GMPs.

Mouse ID	WBC * (x10^{9}/L)	Neutro (x10^{9}/L)	Mono (x10^{9}/L)	Lymph (x10^{9}/L)	RBC (x10^{12}/L)	MCV (fl)	RDW (%)	PLT (x10^{9}/L)
**CMP recipients**								
1	8.7	5.9	0.0	2.7	8.2	37.0	19.2	150
2	2.4	1.8	0.0	0.6	9.5	37.0	17.6	408
3	ND	ND	ND	ND	ND	ND	ND	ND
4	3.8	3.01	0.3	0.5	8.1	41.0	23.5	44
5	ND	ND	ND	ND	ND	ND	ND	ND
6	7.2	3.2	0.0	4.0	6.9	42.0	18.7	471
7	4.0	1.3	0.2	2.5	3.7	51.0	25.0	8
8	4.2	3.0	0.0	1.1	3.5	51.0	24.7	25
9	5.3	2.3	0.0	2.9	9.2	38.0	19.7	155
10	2.8	0.8	0.3	1.7	8.1	37.0	18.9	391
**GMP recipients**								
11	ND[‡]	ND	ND	ND	ND	ND	ND	ND
12	3.1	1.32	0.0	1.73	7.9	37	22.9	146
13	13.6	3.3	0.9	9.4	5.7	36	18.7	216
14	7.7	1.8	0.3	5.6	9.6	37.0	19.9	186
15	5.7	2.3	0.6	2.8	7.0	43.0	17.4	116
16	15.6	7.7	0.7	7.2	8.6	36.0	20.6	381
17	1.9	0.9	0.0	1.0	5.8	45.0	20.5	398
18	ND	ND	ND	ND	ND	ND	ND	ND
19	ND	ND	ND	ND	ND	ND	ND	ND
20	ND	ND	ND	ND	ND	ND	ND	ND
21	3.5	2.6	0.0	0.8	8.1	42.0	18.1	258
22	3.5	2.4	0.4	0.7	10.4	37.0	21.0	423
23	10.9	8.0	0.5	2.4	3.2	55.0	20.6	599
24	18.5	11.1	2.3	5.2	6.7	49.0	18.9	13

This analysis was performed at the same time and on the same animals as described in **S3 Table**.
MCV = mean corpuscular volume; RDW = red cell distribution width.
* CBC was not done (ND) when a blot clot had formed or the VetScan HM2™ machine was unavailable.
